# ALDH2 Mediates 5-Nitrofuran Activity in Multiple Species

**DOI:** 10.1016/j.chembiol.2012.05.017

**Published:** 2012-07-27

**Authors:** Linna Zhou, Hironori Ishizaki, Michaela Spitzer, Kerrie L. Taylor, Nicholas D. Temperley, Stephen L. Johnson, Paul Brear, Philippe Gautier, Zhiqiang Zeng, Amy Mitchell, Vikram Narayan, Ewan M. McNeil, David W. Melton, Terry K. Smith, Mike Tyers, Nicholas J. Westwood, E. Elizabeth Patton

**Affiliations:** 1 School of Chemistry and Biomedical Sciences Research Complex, University of St. Andrews and EaStCHEM, St. Andrews, Fife, Scotland KY16 9ST, UK; 2 Institute of Genetics and Molecular Medicine, The University of Edinburgh, Crewe Road South, Edinburgh, EH4 2XR, Scotland, UK; 3 MRC Human Genetics Unit, The University of Edinburgh, Crewe Road South, Edinburgh, EH4 2XR, Scotland, UK; 4 Edinburgh Cancer Research Centre, The University of Edinburgh, Crewe Road South, Edinburgh, EH4 2XR, Scotland, UK; 5 Wellcome Trust Centre for Cell Biology, University of Edinburgh, Michael Swann Building, King’s Buildings, Mayfield Road, Edinburgh, EH9 3JR, UK; 6 Department of Genetics, Washington University Medical School, 4566 Scott Avenue, St. Louis, MO 63110, USA; 7 School of Biology, University of St. Andrews, Fife, Scotland KY16 9ST, UK

## Abstract

Understanding how drugs work in vivo is critical for drug design and for maximizing the potential of currently available drugs. 5-nitrofurans are a class of prodrugs widely used to treat bacterial and trypanosome infections, but despite relative specificity, 5-nitrofurans often cause serious toxic side effects in people. Here, we use yeast and zebrafish, as well as human in vitro systems, to assess the biological activity of 5-nitrofurans, and we identify a conserved interaction between aldehyde dehydrogenase (ALDH) 2 and 5-nitrofurans across these species. In addition, we show that the activity of nifurtimox, a 5-nitrofuran anti-trypanosome prodrug, is dependent on zebrafish Aldh2 and is a substrate for human ALDH2. This study reveals a conserved and biologically relevant ALDH2-5-nitrofuran interaction that may have important implications for managing the toxicity of 5-nitrofuran treatment.

## Graphical abstract

**Graphical Abstract:**

**Figure:**
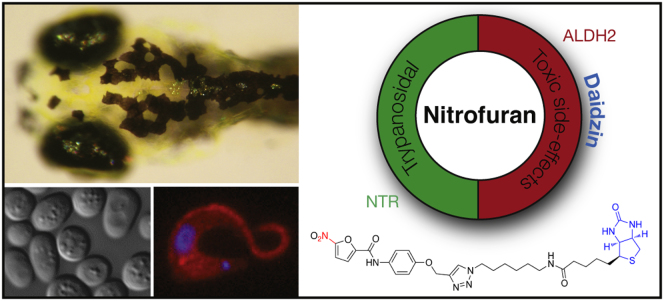

**Highlights:**

► Zebrafish provide a viable assay for the biological toxicity of 5-nitrofurans ► ALDH2 inhibitors prevent 5-nitrofuran toxicity in zebrafish and yeast ► Genetic dependence on ALDH2 for 5-nitrofuran toxicity in zebrafish and yeast systems ► 5-Nitrofurans bind to and are substrates of human ALDH2

## Teaser

5-nitrofurans are antibiotics activated by pathogen specific enzymes, however, less is known about what happens in the host. Zhou et al. identify aldehyde dehydrogenase 2 as a 5-nitrofuran activating enzyme that has implications for managing some of the toxicity associated with 5-nitrofuran treatment.

## Introduction

Drugs often have multiple targets in vivo that can lead to unintended side effects. Identifying unintended drug targets and their in vivo relevance is a fundamental challenge in chemical biology. 5-Nitrofurans are a class of drugs that save thousands of lives as front-line treatments for parasitic trypanosome infections in Latin America and Africa, and they are also effective antibiotics in human and veterinary medicine (Castro et al., 2006; Coura and Viñas, 2010; Nussbaum et al., 2010; Priotto et al., 2009). 5-Nitrofurans are of such importance to human health that the World Heath Organization deems the 5-nitrofuran, nifurtimox, an essential medicine and Bayer HealthCare provides nifurtimox free of charge for trypanosome infections. 5-Nitrofurans are prodrugs, and their relative specificity comes from parasitic and bacteria-specific nitroreductases (NTRs) that reduce the 5-NO_2_ functional group to a toxic anion radical, thereby generating reactive oxygen species and inducing cell death. Despite their widespread use, 5-nitrofurans have serious toxic side effects (Castro et al., 2006). For nifurtimox, toxic side effects lead to treatment cessation in over 30% of patients with Chagas disease, which is caused by *Trypanosoma cruzi* infection (Castro et al., 2006). Clinical side effects are complex and can vary between populations, but they include polyneuropathy, depression, forgetfulness, alcohol intolerance, and headaches, as well as gastrointestinal complications. There is currently no treatment strategy available to reduce the off-target toxic side effects of 5-nitrofurans.

Over decades of research, scientists have identified multiple human enzymes capable of 5-nitrofuran reduction in vitro, in cells or tissues (Dubuisson et al., 2001; Rao et al., 1987; Rao and Mason, 1987). However, the question of whether these enzymes are relevant to 5-nitrofuran side-effect activity and the potential for therapeutic intervention to inhibit their off-target activity in vivo is unanswered. Drug mechanism of action is readily examined in the zebrafish model system, in which clinically active compounds can be directly assayed in the transparent embryo (Zon and Peterson, 2005). Within 2 to 5 days of development in zebrafish, most tissues and organs have formed, thereby enabling the identification of tissue-specific drug activities and/or bioactivation. These features allow facile phenotypic chemical screens within the whole animal. Phenotypic small-molecule screens in zebrafish have enabled the identification of new biological pathways, novel bioactive chemicals, and unexpected potential for known drugs (Taylor et al., 2010). Drugs often have multiple targets in vivo, and examining the effects of small molecules on the developing zebrafish can also identify unintended drug targets (Ishizaki et al., 2010; Ito et al., 2010; Laggner et al., 2012; Rihel et al., 2010).

Here, we use a multispecies approach to identify ALDH2 as a mediator of 5-nitrofuran toxicity in yeast and zebrafish, and we show that 5-nitrofurans are substrates for human ALDH2 in vitro. In a zebrafish phenotypic screen, we found that 5-nitrofurans are melanocytotoxic. We exploited this highly visible in vivo activity to generate a 5-nitrofuran probe, identify ALDH2 as a 5-nitrofuran target, and validate the interaction in vivo. This interaction is conserved from yeast to human, and is also relevant for the clinically active 5-nitrofuran nifurtimox. We propose that this new interaction may be relevant to some of the 5-nitrofuran toxicity observed in the clinic.

## Results

### 5-Nitrofurans Are Active in Zebrafish

**Figure 1:**
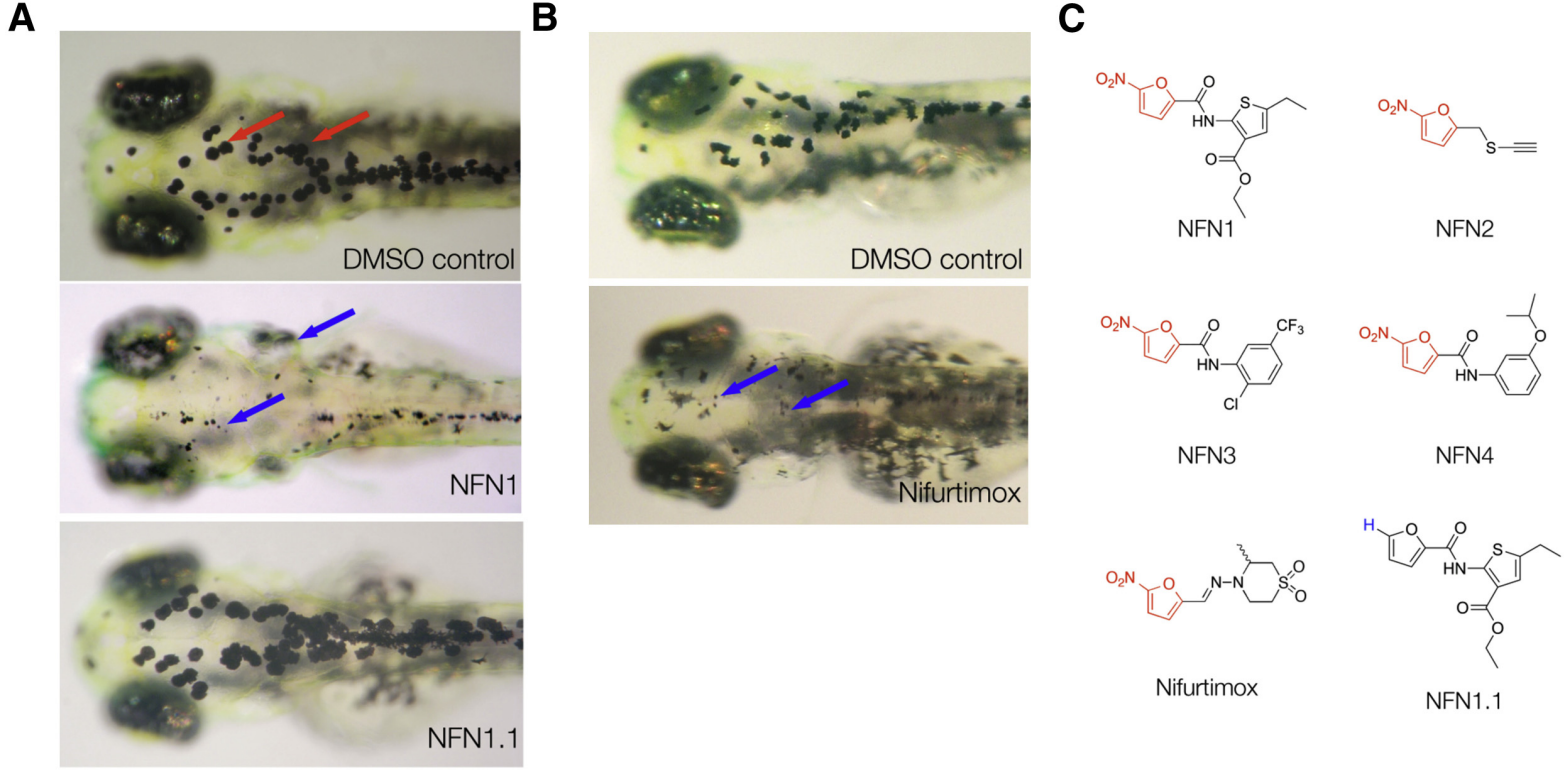
5-Nitrofurans Promote Melanocytotoxicity in Zebrafish (A and B) Examples of zebrafish embryos treated at 2 dpf for 48 hr with DMSO as a control, plus 5 μM NFN1 and 5 μM NFN1.1 (A) or 50 μM nifurtimox (B). Black melanocytes (red arrows) and melanocyte detritus (blue arrows) are indicated. (C) Chemical structures of the four 5-nitrofurans (NFN1–4 [Maybridge compounds BTB05727, SEW00138, BTB13657, and BR00087]) identified in a chemical screen for modulators of melanocyte development. The 5-NO_2_-furan functional group shared between the 5-nitrofurans, including nifurtimox, is indicated in red. The chemical structure of NFN1.1. is identical to that of NFN1 but lacks the 5-NO_2_ functional group required for activity (blue). See also Figure S1 and Movie S1.

Melanocytes are pigment-producing cells that generate black melanin, and pigmented melanocytes are clearly visible in the developing zebrafish beginning at 28 hr postfertilization (hpf; Figure 1A). We identified four 5-nitrofuran compounds, NFN1 (Maybridge BTB05727), NFN2 (SEW00138), NFN3 (BTB13657), and NFN4 (BR00087), in a chemical screen for modulators of melanocyte development in zebrafish embryos (Figures 1A and 1C; see Methods). We also found that zebrafish were sensitive to the clinically active 5-nitrofuran nifurtimox (Figures 1B and 1C). 5-Nitrofuran treatment directly affected the melanocyte and melanocyte progenitor viability in a dose-dependent manner and was independent of tyrosinase activity (Figure S1 available online; Movie S1). Thus, 5-nitrofurans are melanocytotoxic in zebrafish, and unlike prodrugs that are bioactivated by pigmentation enzymes (Jawaid et al., 2009; Yang and Johnson, 2006), their activity is independent of tyrosinase. Altered pigmentation is not a feature of 5-nitrofuran toxicity in humans, but melanocyte specificity in zebrafish provided a rapid, convenient, and highly visible assay to study 5-nitrofuran activity in an animal model, independent of trypanosome infection.

### 5-Nitrofuran Activity Requires the 5-NO_2_ Moiety

5-Nitrofurans are prodrugs, and the 5-NO_2_ moiety is essential for bioactivation in parasites and bacteria (Maya et al., 2007). We modified NFN1 by replacing the NO_2_ moiety with a hydrogen atom (Figure 1C, NFN1.1; Table 1; Supplemental Information). In contrast to treatment with NFN1, NFN1.1 had no effect on zebrafish melanocytes, and the melanocyte remained pigmented and intact (Figure 1A; Table 1). Nitrofuran activity in melanocytes is therefore dependent upon the 5-NO_2_ functional group. As in humans, zebrafish do not have NTRs (which are present in trypanosomes) to process the 5-NO_2_ functional group, and thus, the effects of NFN1 on zebrafish melanocytes may provide information about alternative methods of 5-nitrofuran processing.

### Nitrofurans Bind ALDH2 in Zebrafish

**Figure 2:**
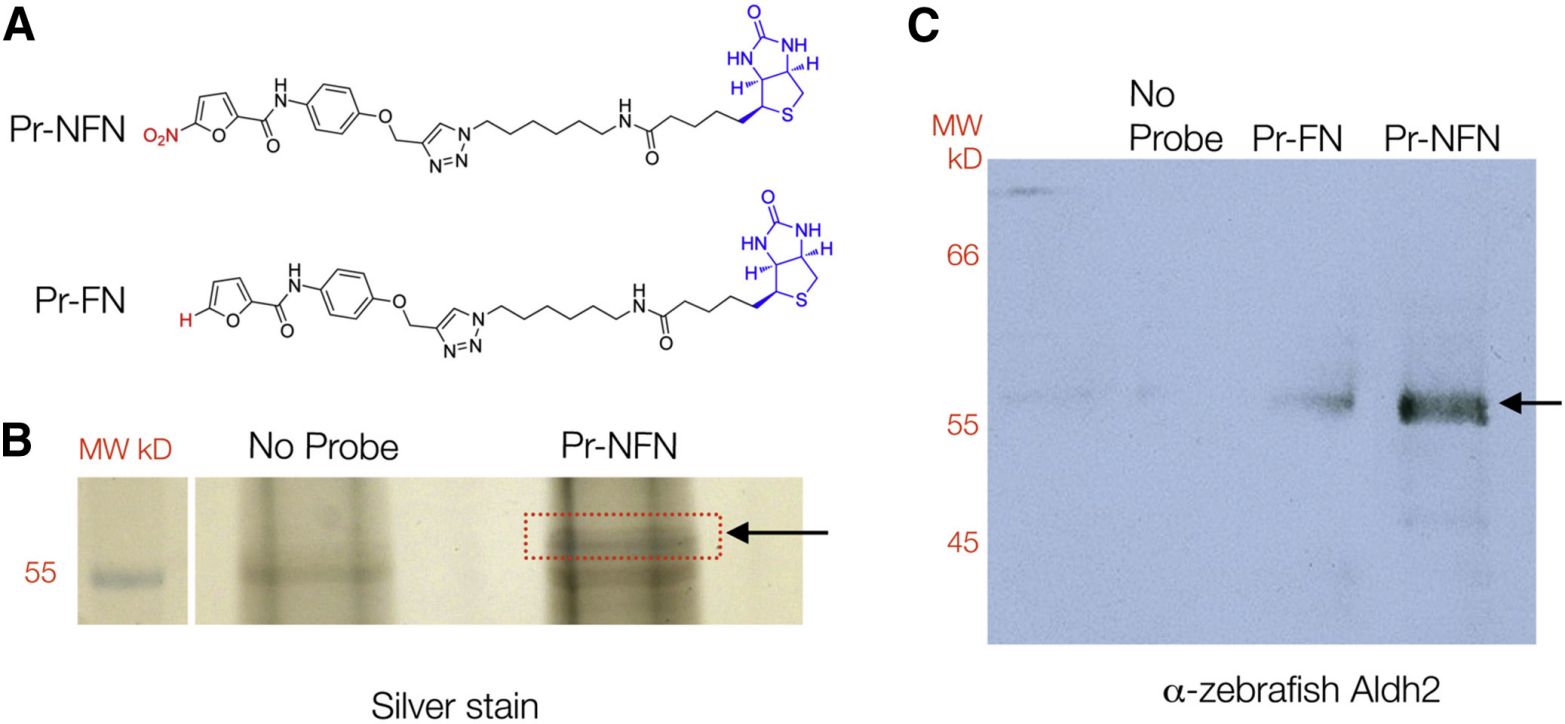
5-Nitrofurans Bind Aldh2 in Zebrafish (A) Biotinylated probes linked to a 5-nitrofuran (Pr-NFN) and a control furan (Pr-FN). Biotin is labeled in blue and the 5-nitro or modification moiety in red. (B) Silver stain of protein bands identified using Pr-NFN probe, or streptavidin beads alone as a control (No Probe). The red box indicates the region of the gel that was isolated for mass spectrometry analysis (arrow) at 57 kD. (C) Western blot of zebrafish protein bound to the no-probe control, the furan (Pr-FN) control, or the 5-nitrofuran probe (Pr-NFN), and probed with zebrafish anti-Aldh2 antibodies. A band corresponding to 57 kDa is indicated (arrow). MW, molecular weight. See also Figure S2 and Table S1.

To identify the possible targets of the 5-nitrofurans, we performed affinity purification to capture 5-nitrofuran interacting proteins in zebrafish extracts. First, we generated a series of 5-nitrofuran derivatives and tested their activity in zebrafish (Table 1; Supplemental Information). Importantly, 5-nitrofuran derivatives containing a phenyl ring (NFN5, NFN5.1, NFN5.2) effectively targeted zebrafish melanocytes (Table 1). As substitution at the *para* position of the phenyl ring in NFN5.1 and NFN5.2 was tolerated, a 5-nitrofuran probe was generated by linking to biotin through the *para* position of the phenyl ring (Pr-NFN; Figure 2A). Next, the 5-nitrofuran probe was bound to streptavidin beads, and protein complexes captured from zebrafish extract derived from 3-day embryos were subjected to tandem mass spectrometry. A 57-kD binding protein was identified as aldehyde dehydrogenase (Aldh) 2b (Figure 2B; Table S1). Zebrafish have two *aldh2* (Lassen et al., 2005; Song et al., 2006) genes (*a* and *b*) that are orthologs of human ALDH2 (Figure S2); *aldh2b* is expressed in neural crest derived cells, including presumptive melanocytes (Thisse et al., 2001). To confirm the identity of the 57-kD protein, we repeated our affinity purification protocol and performed western blotting with anti-Aldh2 zebrafish antibodies raised against both a and b forms of Aldh2 (Lassen et al., 2005) (Figure 2C). As a control, we generated a furan probe that was identical to the nitrofuran probe except that it lacked the 5-NO_2_ functional group (Pr-FN; Figure 2A). Aldh2 (either a or b) bound more strongly to the 5-nitrofuran probe than to the control probe, and not to streptavidin beads alone (Figure 2C). These experiments validate Aldh2 as a 5-nitrofuran binding protein.

### Aldh2 Is Required for 5-Nitrofuran Activity in Zebrafish

**Figure 3:**
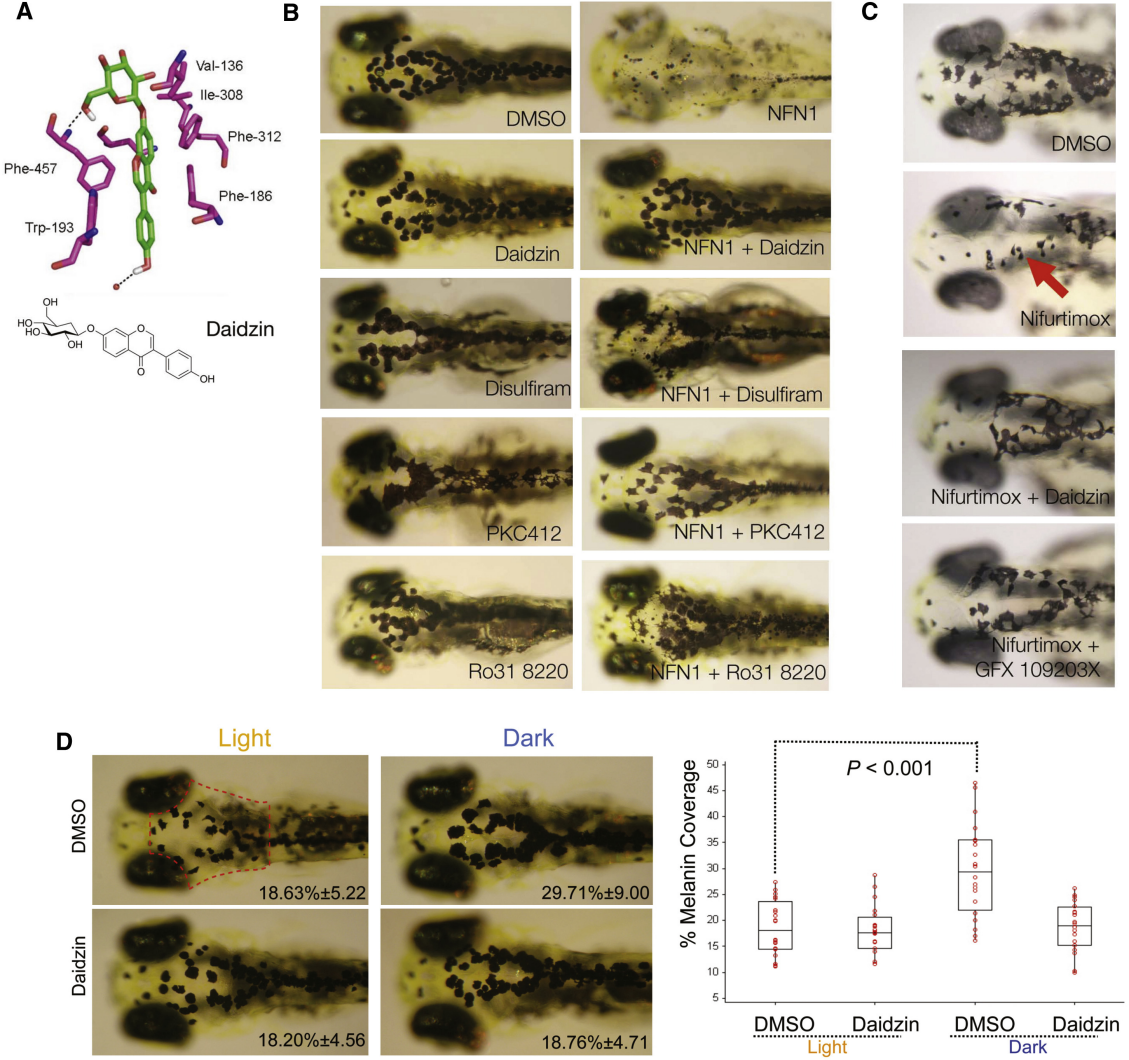
Aldh2 Is Responsible for 5-Nitrofuran Activity in Zebrafish (A) A predicted model of daidzin binding to zebrafish ALDH2b, based on key residues involved in the human ALDH2-daidzin (PDB 2vle) protein-ligand interaction (Lowe et al., 2008). The equivalent residues in zebrafish Aldh2b are shown. Human ALDH2→Zebrafish Aldh2 2b (Phe-459→Phe-457; Phe-170→Phe-186; Trp-177→Trp-193; Val-120→Val-136; Phe-296→Phe-312; Phe-292→Ile-308; Asp-457→Asn-473; Cys-303→Cys-319). (B) Aldh2 and PKC inhibitors prevent 5-nitrofuran activity in zebrafish. Examples of zebrafish embryos treated at 2 dpf with 20 μM of the ALDH inhibitors daidzin or DSF for 1 hr, or with 20 μM of the PKC inhibitors PKC412 or Ro318220, and then treated with 5 μM NFN1 or 0.1% DMSO alone for 2 days. Experiments were repeated at least three times, with n > 10 embryos per condition. (C) Examples of 2 dpf zebrafish embryos pretreated with DMSO, 30 μM of daidzin, or the PKC inhibitor GFX 109203X for 1 hr, and then treated with 50 μM nifurtimox for 7 hr. Punctate melanocytes are indicated. Experiments were repeated at least three times (n = 5–10 embryos per condition) and treatment-condition cohorts blind scored. (D) Daidzin alters background adaptation in zebrafish embryos. (Left) Images of fixed zebrafish embryos (5 dpf) treated with 0.1% DMSO or 10 μM daidzin, and shifted from a dark environment to a light environment (light), or vice versa (dark). The average percentage of melanin coverage (within the area indicated by the red dotted outline) for each treatment condition ± SD is indicated. (Right) Box plot of melanin coverage (y axis) for each embryo in different treatment conditions (x axis). Individual values taken from one of three experiments are shown as red circles. The box depicts the lower quartile and the upper quartile, with the median depicted by the intersecting line. Whiskers extend between the minimum and maximum of all the data. In DMSO-treated embryos, melanocytes are significantly contracted in the light and expanded in the dark (p < 0.001, n = 20 for each condition; ANOVA, 95% confidence interval [CI] 11.081[5.966, 16.195]). Zebrafish treated with daidzin contract their melanin in response to light environment but do not significantly expand their melanin in response to dark environments (95% CI 0.563[−4.552, 5.677]). The experiment was repeated three separate times with embryos at 5 dpf (n = 5–20 embryos per condition) and once with embryos at 4 dpf (n = 10 embryos per condition). See also Figure S3.

Aldh2 catabolizes toxic aldehydes in the liver after alcohol consumption (Druesne-Pecollo et al., 2009), in the heart after ischemia (Chen et al., 2008), and in dopamine metabolism (Yao et al., 2010). We asked if 5-nitrofuran toxicity was dependent on Aldh2 in zebrafish. The natural product daidzin, found in the Kudzu vine (*Pueraria lobata*), is a potent and specific inhibitor of human ALDH2 and has long been used in traditional medicines as an antidipsotropic (Keung and Vallee, 1993a, 1993b; Lowe et al., 2008). More recently, ALDH2 inhibitors have been shown to reduce anxiety associated with treatment of cocaine and alcohol addiction (Arolfo et al., 2009; Yao et al., 2010). We reasoned that ALDH2 inhibitors were likely to prevent the toxicity of 5-nitrofurans in zebrafish because (1) human ALDH2 is closely related to zebrafish Aldh2 (a and b forms) (Figure S2), and (2) computational modeling of zebrafish Aldh2b bound to daidzin suggests that critical drug-protein interactions are conserved between species (Figure 3A). Treatment of zebrafish embryos with daidzin protected melanocytes from the cytotoxicity of the coadministered 5-nitrofuran NFN1 (Figure 3B), as well as the clinically active 5-nitrofuran nifurtimox (Figure 3C). Thus, coadministration of the Aldh2 inhibitor daidzin abrogates the activity of NFN1 and nifurtimox in zebrafish.

To provide additional evidence that the action of daidzin was by inhibition of Aldh2 and not an additional unintended target, zebrafish embryos were cotreated with NFN1 and a second ALDH1/2 inhibitor, disulfiram (DSF). DSF, also called Antabuse and Antabus, is used to treat chronic alcoholism by preventing the ALDH2-dependent metabolism of alcohol and producing enhanced sensitivity to alcohol. DSF also chelates copper, and we and others have found that DSF prevents pigmentation of zebrafish melanocytes prior to melaninization, most likely due to inhibition of copper-dependent pigmentation enzymes (Figure S3; O’Reilly-Pol and Johnson, 2008). DSF treatment of embryos 3 days postfertilization (dpf) that had fully pigmented melanocytes had no effect on melanocyte integrity, while DSF prevented melanocyte toxicity upon cotreatment with NFN1 (Figure 3B). Taken together, these experiments with two chemically independent ALDH2 inhibitors support a biological role for Aldh2 in the bioactivation of 5-nitrofuran melanocytotoxicity in zebrafish.

*ALDH2* is regulated in a tissue-specific manner, and in particular, εPKC can directly modulate ALDH2 during ischemic preconditioning in the heart (Chen et al., 2008, 2010). We identified the PKC inhibitors PKC412 and Ro318220 as chemical suppressors of 5-nitrofuran activity in zebrafish by screening a library of 80 known kinase inhibitors. Treatment of 3 dpf zebrafish embryos with PKC412 or Ro318220 had no effect on melanocyte viability (Figure 3B). However, treatment with PKC412 or Ro318220 prevented NFN1 activity in melanocytes (Figure 3B). We tested a third PKC inhibitor, GF109203X, that can inhibit ethanol or dopamine D2 receptor agonist NPA-induced intracellular translocation of εPKC (Yao et al., 2008). GFX109203X had no effect on melanocytes alone, but we found that it could also suppress NFN1 melanocytotoxicity (Figure S3). GFX109203X was also effective at preventing the activity of nifurtimox in zebrafish melanocytes (Figure 3C). Although we do not know if PKC directly enhances Aldh2b activity or expression in zebrafish, these results suggest that PKC activity is important for 5-nitrofuran cytotoxicity within the melanocyte.

### ALDH2 Contributes to Background Adaptation in Zebrafish Melanocytes

We wanted to understand why zebrafish melanocytes were sensitive to 5-nitrofuran treatment, when this is not a feature of 5-nitrofuran toxicity in patients. Unlike human melanocytes, zebrafish melanocytes respond to environmental conditions by concentrating or dispersing their melanosomes in light or dark conditions, respectively (Logan et al., 2006). This effect is termed background adaptation and is a dopaminergic response (Logan et al., 2006). A role of Aldh2 in zebrafish background adaptation has not been previously identified, but *aldh2b* is specifically expressed in developing pigment cells (Thisse et al., 2001), and ALDH2 is required for dopamine metabolism in mammals (Chen et al., 2010). We tested the effects of ALDH2 inhibition on background adapation in zebrafish and found that daidzin treatment blocked dispersal of melanin in zebrafish melanocytes in the dark (Figure 3D). These observations suggest that Aldh2 activity is required for regulation of zebrafish background adaptation, and they may explain the sensitivity of zebrafish melanocytes to 5-nitrofurans.

### Multispecies Conservation of the 5-Nitrofuran-ALDH Interaction

**Figure 4:**
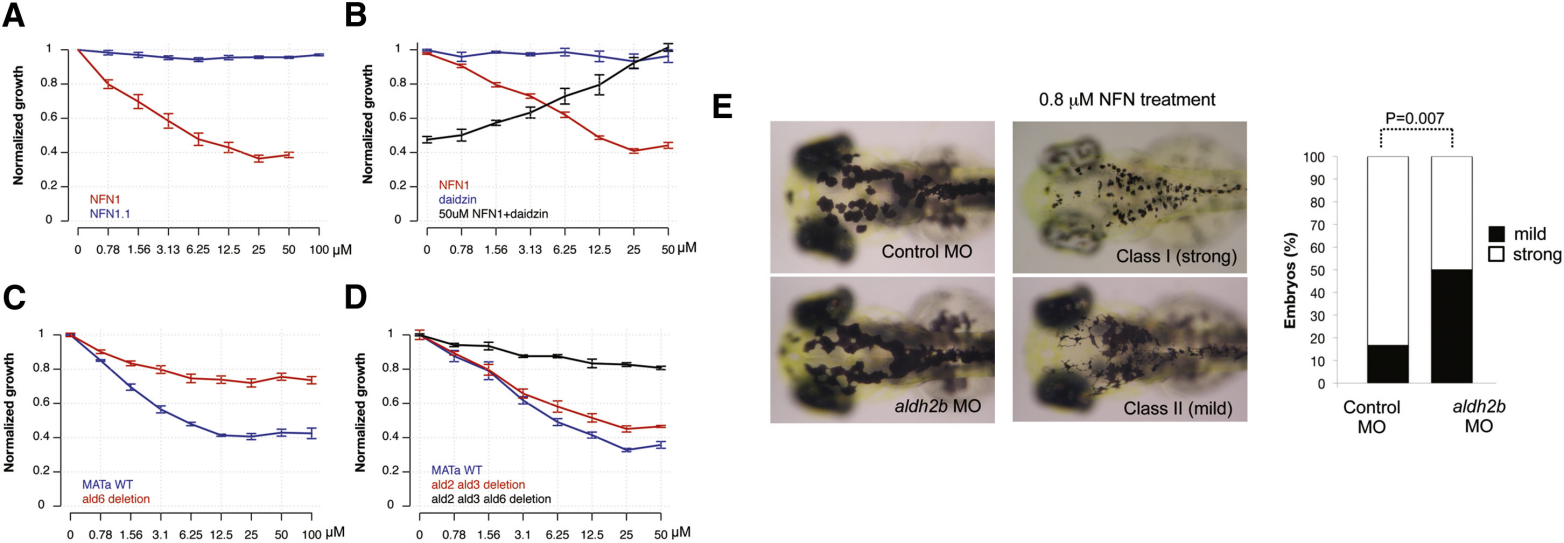
Cross-Species Conservation of 5-Nitrofuran-ALDH2 Interaction in Yeast (A) Yeast cultures were treated with NFN1 (red) or NFN1.1 (blue). OD values were normalized against DMSO-treated controls. The mean of two experiments with three replicates is shown; error bars represent the SE. (B) Daidzin-NFN1 drug interaction was assessed by combination matrix assays in 96 well plates. Cultures were treated with NFN1 (red) or with daidzin in the absence (blue) or presence (black) of 50 μM NFN1. The average normalized growth of three experiments is shown; error bars represent the SE. (C) Normalized growth in the presence of NFN1 was determined for wild-type (blue) and the *Δald6* strain (red). Data points are the mean of four replicates; error bars represent the SE. (D) NFN1 dose response curves for Δ*ald2*Δ*ald3* (red) and the Δ*ald2*Δ*ald3Δald6* (black) strains, as well as wild-type control (blue), were generated and normalized against DMSO-treated controls. The average of three replicates is shown; error bars represent the SE. (E) Control (n = 24) or *aldh2b* splice-site morphants (n = 62) at 3 dpf without NFN1 treatment (left) or with 0.8 μM NFN1 treatment (right). Embryos were scored as class I (strong) or class II (mild) sensitivity to NFN1 (bar graph). *aldh2b* morphant embryos were less sensitive to NFN1 treatment compared to control morphants (p = 0.007; 95% CI [0.139, 0.528]; Fisher’s exact test). See also Figure S4.

Chemical-genetic and chemical-chemical interactions identified in yeast are often conserved in multicellular species including zebrafish and mammals (Ishizaki et al., 2010). Budding yeast have five ALDH genes (*ALD2–6*) that all share 42%–48% similarity with human *ALDH 1/2* (Figure S2). Yeast also have two fungal-specific nitroreductase-like proteins, but these share little similarity with the nitroreductases that are known to reduce nitrofurans (de Oliveira et al., 2007). To establish that 5-nitrofurans also showed activity in yeast, liquid cultures were treated with increasing concentrations of NFN1 (Figure 4A). Yeast were highly sensitive to NFN1, which inhibited growth even at submicromolar concentrations. In contrast, treatment with the control furan compound, NFN1.1, had no effect on yeast growth, even at 100 μM. These data indicate that the toxicity of 5-nitrofurans in yeast is dependent on the 5-NO_2_ moiety. To test whether NFN1 toxicity was dependent on ALDH activity, we tested drug combinations in yeast cultures. Increasing concentrations of daidzin rescued the effects of 50 μM NFN1 on the yeast growth rate in a dose-dependent fashion, whereas daidzin alone had no effect on growth (Figure 4B).

Mutations that render yeast resistant to a specific compound can provide direct links to the target pathway (Ishizaki et al., 2010). We determined whether yeast strains bearing deletions in each of the *ALD* genes (orthologs of human and zebrafish *ALDH1/2*) were resistant to 5-nitrofuran treatment. The *ald2*Δ, *ald3*Δ, *ald4*Δ, and *ald5*Δ deletion strains each exhibited the same sensitivity to NFN1 as wild-type (data not shown). In contrast, an *ald6*Δ strain was significantly less sensitive to NFN1 treatment, as was an *ald2*Δ*ald3*Δ double-deletion strain (Figures 4C and 4D). These effects of different *ald* mutations appeared to be additive, as a triple *ald2*Δ*ald3*Δ*ald6*Δ deletion strain was almost completely resistant to 50 μM NFN1 treatment (Figure 4D). Once activated, 5-nitrofurans cause DNA damage, and consistent with this observation, we find that chemical-genetic profiles in yeast indicate that disruption of DNA damage repair pathways causes hypersensitivity to 5-nitrofurans (Figure S4).

To further validate the genetic dependence of 5-nitrofuran bioactivity on Aldh2, we used morpholino oligonucleotides (MOs) to knockdown *aldh2b* in zebrafish. Single-cell embryos were injected with a splice-site-blocking *aldh2b* MO and at 2 dpf were treated with NFN1. PCR analysis of the splice-site MO indicated that *aldh2b* morphants had reduced levels of correctly spliced *aldh2b* transcript in addition to a misspliced transcript, indicating that the *aldh2b* morphants are hypomorphic for *aldh2b* (Figure S4). We consistently found that the splice-site-blocking *aldh2b* MO conferred partial resistance to a low treatment dose (0.8 μM) of NFN1 melanocytotoxicity (Figure 4E). An *aldh2b*-translation-block MO also conferred partial resistance to a short NFN1 treatment (Figure S4). We conclude that there is a genetic dependence on Aldh2b for 5-nitrofuran activation in zebrafish, in line with genetic studies in yeast.

### 5-Nitrofurans Are Substrates for Human ALDH2

**Figure 5:**
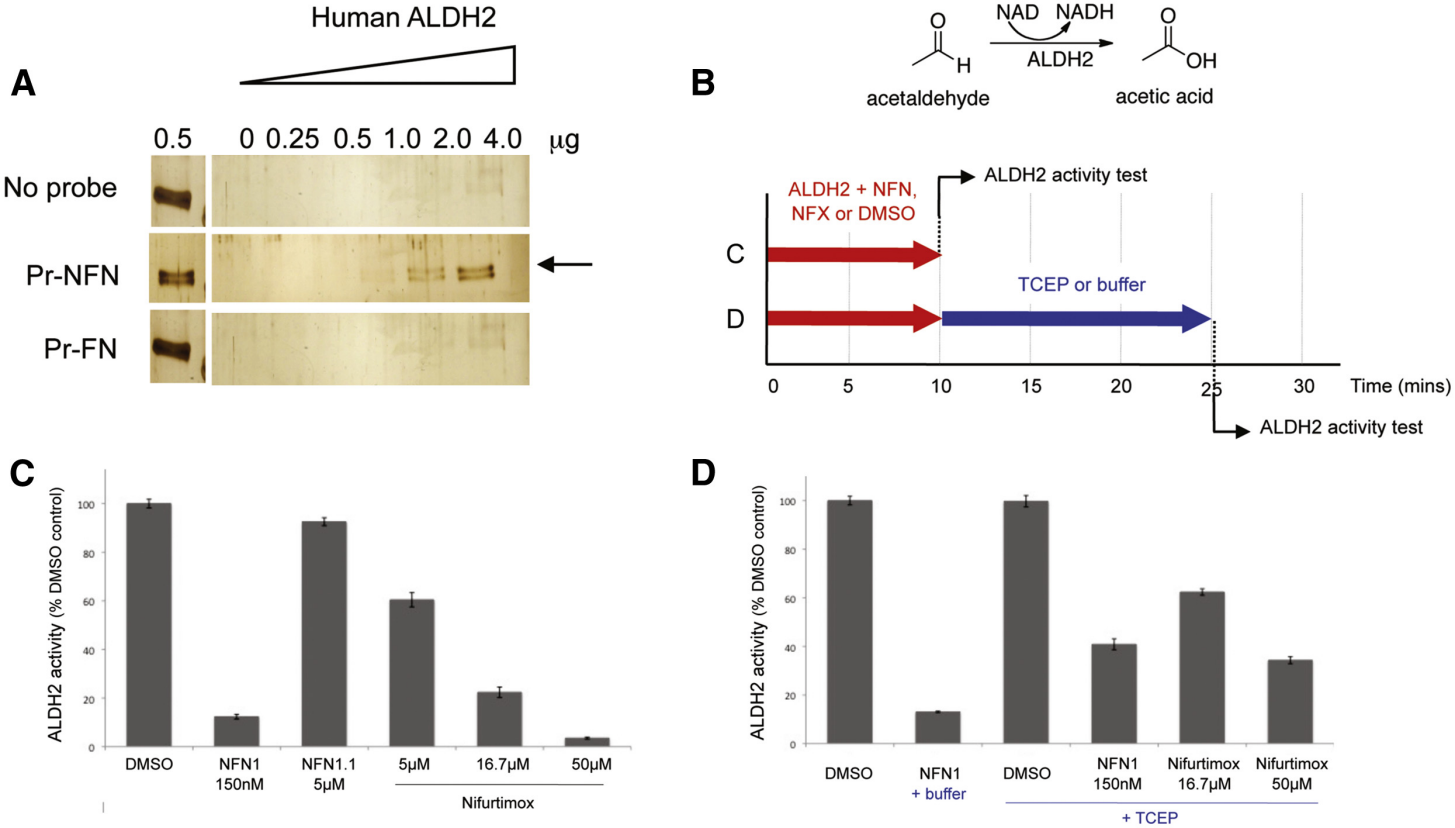
5-Nitrofurans Bind and Are Substrates for Human ALDH2 In Vitro (A) Binding of purified human ALDH2 by 5-nitrofuran probe (Pr-NFN), a furan control probe (Pr-FN), or streptavidin beads alone (No Probe). Arrow indicates ALDH2 protein, ALDH2 input lane (0.5 μg). (B) Schematic overview of chemical reaction used to monitor recombinant human ALDH2 activity and experimental design. In experiment C (red arrow), ALDH2 was incubated with 1% DMSO, NFN1, and NFN1.1 or Nifurtimox for 10 min., and then ALDH2 activity was assessed. In experiment D (red + blue arrows), ALDH2 was incubated with 1% DMSO, NFN1, or Nifurtimox for 10 min., incubated with 0.5 mM TCEP or buffer alone for a further 15 min., and then ALDH2 activity was assessed. (C) Bar graph of spectrophotometric analysis of the rate of production of NADH (monitored at 341 nm) by ALDH2 (expressed as a percentage of DMSO control treatment) with DMSO, NFN1, NFN1.1, and Nifurtimox. (D) Bar graph of spectrophotometric analysis of the rate of production of NADH by ALDH2 after combined treatment of DMSO, NFN1, and Nifurtimox with TCEP or buffer. Enzyme buffer = 50 mM sodium phosphate (pH 7.4). Error bars are SD; experiments were repeated in triplicate.

There are 19 ALDH enzymes in humans, each with specific targets and additional activities (Marchitti et al., 2008). To determine whether the 5-nitrofuran-ALDH2 interaction is conserved in humans we asked whether human ALDH2 could bind 5-nitrofurans directly. Purified human ALDH2 was added to the 5-nitrofuran probe (Pr-NFN), a furan control probe (Pr-FN), or streptavidin beads alone. In an analogous manner to the experiments using zebrafish extracts, human ALDH2 binding was strongly enriched in association with the 5-nitrofuran, while the control furan and the streptavidin beads alone did not bind ALDH2 (Figure 5A).

Given our results with daidzin in yeast and zebrafish, we proposed that NFN1 was probably a substrate of ALDH enzymes. ALDH2 enzymes have reducing potential as well as dehydrogenase activity (Chen et al., 2002; Marchitti et al., 2008), and it has been shown that in the absence of a reducing agent, ALDH2 inactivates itself during the bioactivation of substrates such as nitroglycerine (GTN) (Chen et al., 2010; Wenzel et al., 2007). Consistent with this, we found that in the absence of a reducing agent, NFN1, but not the no-nitro NFN1.1, inactivated recombinant human ALDH2 in vitro (Figures 5B–5D). Likewise, we found that ALDH2 activity was reduced by 39.6%, 77.6%, and 96.5% following 10 min incubation with 5 μM nifurtimox, 16.7 μM nifurtimox, and 50 μM nifurtimox, respectively (Figure 5C). Importantly, as with the zebrafish studies, these experiments were performed with nifurtimox at concentrations that are within the range of those recorded in the serum of nifurtimox-treated patients (Paulos et al., 1989; Saulnier Sholler et al., 2011). For both NFN1- and nifurtimox-inactivated ALDH2, the subsequent addition of a reducing agent (TCEP) led to partial reactivation of the enzyme, in line with literature studies using the accepted substrate, GTN (Figure 5D). We observe that the NFN1-ALDH2 interaction is stronger than the nifurtimox-ALDH2 in zebrafish and in our biochemical assay. This raises the possibility that the mechanism of action of nifurtimox is more complex than that of NFN1, or that NFN1 may in fact be a more effective 5-nitrofuran substrate of ALDH2 than nifurtimox.

### Daidzin Does Not Affect Nifurtimox Trypanocidal Activity

**Figure 6:**
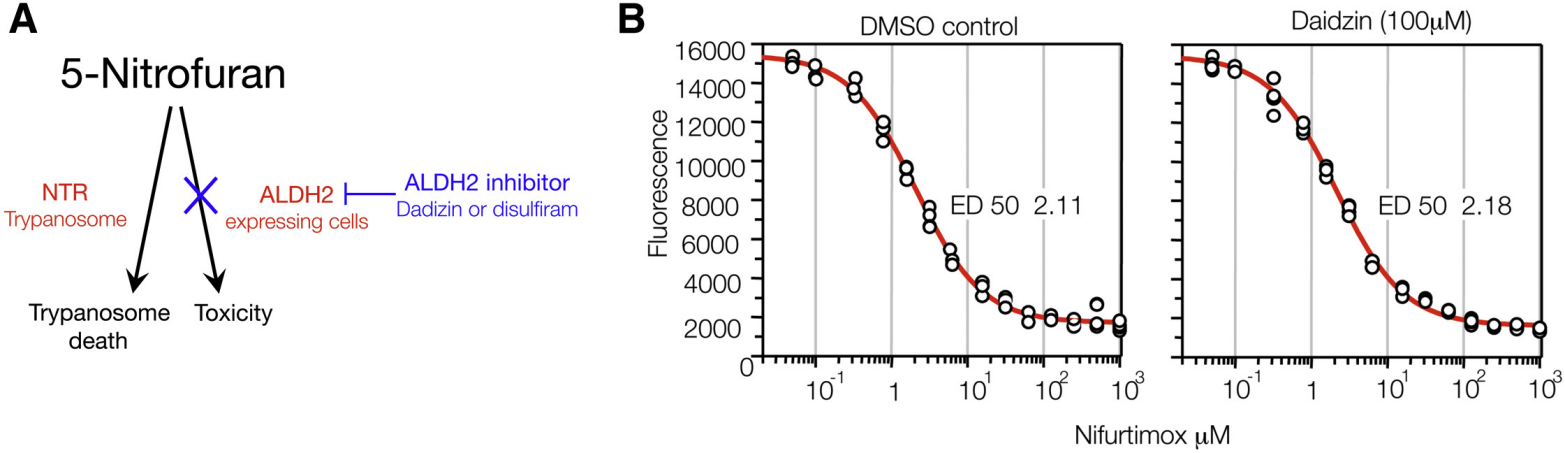
ALDH2 in Trypanosomes (A) Schematic of a 5-nitrofuran-daidzin combination-treatment strategy. ALDH2 can cause 5-nitrofuran bioactivtion in ALDH2-expressing cells (e.g., zebrafish melanocytes), but not in trypanosomes because they lack ALDH2 (see also Figure S2). We propose that cotreatment with an ALDH2 inhibitor such as daidzin could limit 5-nitrofuran toxicity without interfering with antitrypanosome activity. (B) Viability of *Trypanosoma brucei* (bloodstream form) at 37°C after 72 hr treatment with increasing concentrations of nifurtimox in the absence or presence of daidzin (100 μM). Experiments were conducted twice in replicates of four; a representative set of data from one experiment containing four replicates is shown. ED, effective dose.

In an attempt to develop a clinically testable hypothesis, we examined the genome sequence of the trypanosomatids to identify possible ALDH enzymes in *T. brucei*, *T. cruzi* and *Leishmania* (Figure S2) (Aslett et al., 2010; Cross, 2005; Lowe et al., 2008; Marchitti et al., 2008; Sobreira et al., 2011). Given the absence of an obvious ALDH2 in *Trypanosoma* we hypothesized that while Aldh2 inhibition would protect the zebrafish melanocytes and yeast cells from 5-nitrofuran activity, ALDH2 inhibitors might not protect trypanosomes from 5-nitrofuran sensitivity (Figure 6A). We grew the bloodstream-form *T. brucei* (strain 427) in HMI9 media and determined the trypanocidal activity of nifurtimox in the absence and presence of daidzin. Trypanosomes were stained with an Alamar Blue vital dye as an indicator of *Trypanosoma* survival. We found that nifurtimox was equally effective in the absence (ED_50_ = 2.12 ± 0.17 μM; slope 1.00) and presence (ED_50_ = 2.18 ± 0.10 μM; slope 0.98) of daidzin (Figure 6B). The trypanocidal effect of nifurtimox against bloodstream *T. brucei* obtained in these assays was comparable to previously observed effects (Priotto et al., 2009; Sokolova et al., 2010). Daidzin treatment alone showed no trypanocidal effect up to 100 μM (data not shown). We conclude that daidzin does not interfere with 5-nitrofuran trypanocidal activity, consistent with a lack of an *ALDH2* in trypanosomes.

## Discussion

We have used a multispecies, chemical-biology approach to identify 5-nitrofurans as substrates for ALDH2. We have identified a series of 5-nitrofuran compounds by phenotypic screening in zebrafish and have shown that 5-nitrofuran-specific melanocytotoxicity in vivo is mediated at least in part by Aldh2 (Figures 1 and 3). Zebrafish gene products are usually conserved in humans and are often sensitive to clinically active drugs at physiological concentrations (Zon and Peterson, 2005). As shown here, phenotypic chemical screens in zebrafish are effective because (1) the rapid and cell-type-specific toxicity of 5-nitrofurans can be visualized in real time (Movie S1), (2) the whole animal is amenable to pharmacological studies (Figures 1A and 1B), and (3) initial structure activity relationships can be determined to enable the design of biologically relevant probes for affinity purification (Figure 2; Table 1).

Despite the benefits of phenotypic screens in zebrafish, target identification remains a challenge in chemical biology (Laggner et al., 2012; Taylor et al., 2010; Zon and Peterson, 2005). Here, we use parallel approaches to enable identification of an important target of 5-nitrofurans. First, we used affinity chromatography to identify Aldh2 as a 5-nitrofuran binding partner and confirmed the dependence on the 5-NO_2_ functional group using an inactive furan probe (Figure 2). Second, we used computational modeling to predict that the ALDH2 inhibitor daidzin would be active in zebrafish (Figure 3A), and used two chemically distinct ALDH2 inhibitors (daidzin and DSF) to confirm the biological relevance of the 5-nitrofuran-Aldh2 interaction in vivo (Figures 3B and 3C). Third, we showed cross-species conservation of the drug-drug interactions in the evolutionarily distant budding yeast system (Figures 4A and 4B). Fourth, we used genetic mutants in yeast and gene knockdowns in zebrafish to validate a genetic dependence on ALDH activity for 5-nitrofuran activity in vivo (Figures 4C–E). Fifth, we showed that the 5-nitrofuran-ALDH2 interaction is maintained with human ALDH2 (Figure 5A). Finally, using a literature-precedent method, we showed that 5-nitrofurans are direct substrates of human ALDH2 (Figures 5B–5D).

We find that zebrafish melanocytes are sensitive to the 5-nitrofurans because unlike human melanocytes, zebrafish melanocytes use ALDH2 to elicit a melanocyte background adaptation response (camouflage; Figure 3D). While additional host enzymes, possibly including other ALDHs, may bioactivate 5-nitrofurans in patients, we speculate that, in line with our studies in zebrafish and yeast, daidzin may protect cells that specifically express ALDH2, such as the liver and dopaminergic neuronal cells (Figure 6A). Although 500 million individuals worldwide have an ALDH2-inactive variant (Druesne-Pecollo et al., 2009), it is unknown whether these genetic variants contribute to the variability of 5-nitrofuran-associated side effects; our chemical-genetic data in yeast and zebrafish (Figure 4) suggest that this hypothesis could be examined in the clinic. 5-Nitrofurans have also recently become anticancer agents, and nifurtimox is currently in clinical trials for relapsed/refractory pediatric neuroblastoma and medulloblastoma (Saulnier Sholler et al., 2011). It is possible that 5-nitrofuran bioactivation by ALDH2 explains the sensitivity of these dopaminergic cancers to nifurtimox. We find that human melanoma cells are also sensitive to nitrofurans, that DNA damage occurs, and that this activity is dependent on the NO_2_ functional group present in NFN1 (Figure S4). Taken together with the hypersensitivity of yeast DNA-damage mutants to NFN1, these results suggest that once activated, the cytotoxic effects of 5-nitrofurans arise through a similar DNA-damage-dependent mechanism across species, although it is unclear at this time whether NTR- and ALDH2-mediated activation of 5-nitrofurans leads to exactly the same toxic intermediates.

We argue that NFN1, but not the no-nitro NFN1.1, is a substrate for recombinant human ALDH2 in vitro (Figure 5). Analogous observations have been made in ALDH2 bioactivation of nitroglycerin (Chen et al., 2010; Wenzel et al., 2007), thereby raising the interesting question of how 5-nitrofurans are bioactivated by ALDH2. ALDH2 enzymes have reducing potential as well as dehydrogenase activity (Chen et al., 2002; Marchitti et al., 2008), and we envision that ALDH2 may reduce the nitro group of 5-nitrofurans, potentially generating nitroso-, hydroxylamine, and/or amine intermediates with concomitant oxidation of the enzyme. Interestingly, dithiothreitol (DTT) can react with 5-nitrofurans, leading to oxidation of DTT to the corresponding disulfide (L.Z. and N.W., unpublished data). As DTT contains two thiols in close proximity, in an analogous manner to the active site of ALDH2, we suggest that the reactions of 5-nitrofurans with ALDH2 and DTT may be linked by a common mechanism.

5-Nitrofurans are important therapeutic agents, yet many patients suffer from unacceptable drug-induced toxic side effects. One approach to solving this problem is to identify new antitrypanosome drug targets, such as the recently identified *N*-myristoyltransferase inhibitors (Frearson et al., 2010) that have been validated in mouse trypanosomiasis models. Based on our studies in model systems and in vitro, we propose a complementary approach that involves targeting and minimizing the toxic side effects of current therapies, thereby allowing more patients to benefit from approved treatment regimes that are already available (Figure 6A). If the 5-nitrofuran-ALDH2 interaction is conserved in patients, then combination therapy to treat 5-nitrofuran toxic side effects may be testable, because (1) ALDH2 is a targetable enzyme; (2) the ALDH2 inhibitors daidzin and DSF are both currently available at low cost and show activity in humans with limited toxicity; and (3) our analysis indicates that *T. brucei* and *T. cruzi* do not have a close ALDH2 homolog (Figure S2), nor is *T. brucei* protected from nifurtimox by daidzin (Figure 6B). Our findings provide impetus for addressing the role of ALDH2 in 5-nitrofuran activation in the preclinical and clinical setting.

## Significance

**Discovering how drugs work in vivo and identifying unintended drug targets is a fundamental challenge in chemical biology. Nifurtimox is one of only two drugs used to treat Chagas disease, caused by *Trypanosoma cruzi* infection, which is estimated to affect over 10 million people per year and kills between 15,000 and 50,000 annually. Like other 5-nitrofurans, nifurtimox is a prodrug that is activated by parasite-specific nitroreductases to a toxic form. Despite the absence of nitroreductases in humans, 5-nitrofurans cause significant clinical off-target toxic side effects that interfere with patients’ ability to complete the treatment course. There has been no significant improvement in trypanosome disease treatment for 40 years, and there is currently no treatment strategy in patients to reduce the burden of these toxic side effects of existing drugs.**

**Here, we use model organism chemical genetics to explore the basis for this toxicity. We use the zebrafish model (1) to identify toxic effects of 5-nitrofuran compounds; (2) as a platform for structure-activity relationships and target identification; and (3) to show that the toxicity of 5-nitrofurans in zebrafish can be prevented by cotreatment with aldehyde dehydrogenase 2 (ALDH2) inhibitors. We then show that the ALDH2-5-nitrofuran interaction is conserved in yeast and with human ALDH2 and argue that 5-nitrofurans are a direct substrate of human ALDH2. We extend these findings to show that the 5-nitrofuran nifurtimox also has Aldh2-dependent activity in zebrafish, and that it is a direct substrate of human ALDH2. Thus, we show in model systems that drug treatments combining ALDH2 inhibitors with 5-nitrofurans block the 5-nitrofuran unintended biological activity, and we propose that similar treatments based on a readily available combination of inexpensive approved drugs may prevent some of the clinical side effects caused by 5-nitrofurans.**

## Experimental Procedures

### Zebrafish Small-Molecule Screens and Treatments

All zebrafish work was done in accordance with United Kingdom Home Office Animals (Scientific Procedures) Act (1986) and approved by the University of Edinburgh Ethical Review Committee. The chemical library was a collection of 1576 Maybridge compounds (Ishizaki et al., 2010). Two 4 hpf embryos were arrayed in 96 well plates containing 10 μM of compound in 1% DMSO in 300 μl of E3 embryo medium. Embryos were assessed and imaged for phenotypic changes at 28, 36, 48, and 56 hpf. For the screening of The Screen-Well Kinase Inhibitor Library (Enzo Life Sciences), five embryos (24 hpf) were placed into each well of a 24 well plate (Corning) containing 20 μM NFN1 (BTB05727, Maybridge Screening compounds) and 5, 10, or 20 μM of a corresponding compound (total volume 1 ml per well). For cotreatment experiments, five 36–48 hpf embryos were arrayed in 24 well plates in 600 μl to 1 ml of E3 embryo medium and pretreated with ALDH or PKC inhibitors (1–7 hr), and then treated with 0.5–5 μM NFN1 or 50 μM nifurtimox.

### Affinity Purification and Coimmunoprecipitation with 5-Nitrofuran Beads

Lysate was generated from approximately 900 3 dpf zebrafish in 300 μl of RIPA buffer (2 M Tris pH 7.5, 5 M NaCl, 1% NP40, Na-deoxycholate, 10% SDS, 0.5 M NaF, 1 M β-glycosyl phosphate and protease-inhibitor cocktail tablet [Roche]), centrifuged at 4°C (25 min), transferred to a new tube, and kept on ice. Protein capture was performed using a pull-down biotinylated protein:protein interaction kit (Pierce) using the biotinylated chemical probe (5 μl 10 mg/ml DMSO solution), and bead complexes were washed with 0.1 M NaCl TBS buffer four times to reduce nonspecific binding. Beads were boiled in 3× Laemmli buffer with DTT for 5 min and run on 10% SDS-PAGE gel for electrophoresis. Captured proteins were visualized with a Silverquest silver-staining kit and/or Colloidal blue-staining kit (Invitrogen). The mass spectroscopy was analyzed in the University of Dundee FingerPrints Proteomics Facility. For western blotting, protein was detected using rabbit anti-zebrafish Aldh2 (1:1000) and goat anti-rabbit antibody (1.5:5000; Calbiochem).

### In Vitro Binding Assay

ALDH2 human recombinant protein (ProSpec) was added to 4 μl 10 mg/ml of chemical probe with 100 μl TBS buffer and incubated at room temperature for 1 hr. Streptavidin bead suspension (50 μl) was added to the mixture (room temperature; 1 hr), the supernatant was removed, and beads were washed with 4 × 0.1 M NaCl TBS buffer, boiled in 3× Laemmli buffer with DTT for 5 min, and run on 10% SDS-PAGE gel for electrophoresis. The bands were detected by silver staining (Invitrogen).

### Molecular Modeling

Using methods analogous to those used previously (Medda et al., 2009), the zebrafish Aldh2b homology model was generated using the Swiss model server using bovine ALDH2 (PDB code 2AG8). The daidzin structure was generated using the PRODRG server. The docking studies were performed using the program GOLD. All visualization and analysis was performed using Pymol.

### Yeast Growth Assays

Overnight *S. cerevisiae* BY4741 cultures in SC media were diluted (OD_600_ 0.025) and dispensed into 96 well Corning Costar assay plates. Quantitative growth curves were generated in Tecan Sunrise plate readers at 30°C 564 rpm with automated absorbance reads every 15 min. Growth-curve data were used to determine when control cultures reached late log phase, and OD values of the entire plate at that time point were used to calculate normalized growth values. Data were analyzed with custom R scripts to generate plots. For the deletion-strain growth curves, normalization was performed against control wells for each strain.

### Trypanocidal Studies

The trypanocidal activity of nifurtimox in the absence and presence of daidzin (100 μM) against *Trypanosoma brucei* bloodstream form (strain 427) were cultured at 37°C in HMI9 medium supplemented with 2.5 μg ml^−1^ G418, and viability was determined using the Alamar Blue test, as described previously (Mikus and Steverding, 2000). The data were fitted using GraFit software to obtain ED_50_ ± SD and slope factors.

### Supplemental Experimental Procedures

The synthesis of all the NFNs and NFN-based affinity probes is described in the Supplemental Information.

## Figures and Tables

**Table 1:** Derivatives of 5-Nitrofurans and Their Activity in Zebrafish

Compound	0.2 μM	0.4 μM	0.8 μM	1.6 μM
NFN1	No activity	No activity	+	+++
NFN1.1	No activity	No activity	No activity	No activity
NFN5	No activity	+	++	++++
NFN5.1	No activity	+	++	++
NFN5.2	No activity	+	++	++++^a^

+Some melanocytes become dendritic, few are fragmented. ++Some punctate and fragmented melanocytes. +++All melanocytes are punctate, many clearly fragmented, pigment remains in eye. ++++All melanocytes are fragmented, with almost complete loss of pigment in body and eye.

a Additional nonspecific toxicity.

## Supplemental Information

Document 1. Figures S1–S4, Table S1, and Supplemental Experimental Procedures

Movie S1. Time-Lapse Imaging of 5-Nitrofuran Treatment of Live Zebrafish, Related to Figure 1
