# ALDH2 Mediates 5-Nitrofuran Activity in Multiple Species

**DOI:** 10.1016/j.chembiol.2020.10.011

**Published:** 2020-11-19

**Authors:** Linna Zhou, Hironori Ishizaki, Michaela Spitzer, Kerrie L. Taylor, Nicholas D. Temperley, Stephen L. Johnson, Paul Brear, Philippe Gautier, Zhiqiang Zeng, Amy Mitchell, Vikram Narayan, Ewan M. McNeil, David W. Melton, Terry K. Smith, Mike Tyers, Nicholas J. Westwood, E. Elizabeth Patton

## Main Text

(Chemistry & Biology *19*, 883–892; July 27, 2012)

In the originally published version of this article, the No probe control figure panels were inadvertently generated from the Pr-FN control figure panels. The revised Figure 5A appears below with the appropriate panels. The error does not change the interpretation of the data.

The authors regret this error.

**Figure:**
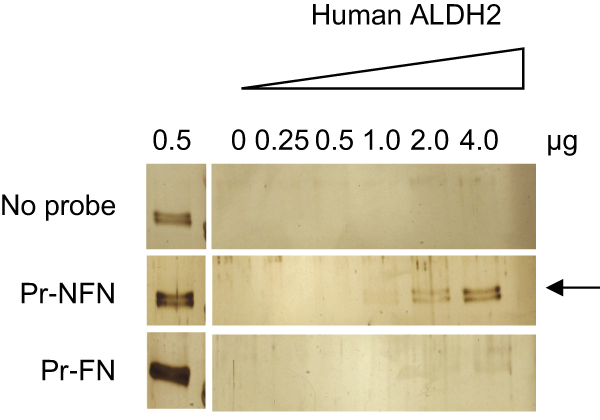
Figure 5A. 5-Nitrofurans Bind and Are Substrates for Human ALDH2 In Vitro

