# Supplementary material for: ALDH2 Mediates 5-Nitrofuran Activity in Multiple Species
Source: Chem Biol. 2012 Jul 27;19(7):883–92. doi: 10.1016/j.chembiol.2012.05.017 (PMC3684953; doi:10.1016/j.chembiol.2012.05.017)

Chemistry & Biology, Volume *19*

Supplemental Information

ALDH2 Mediates 5-Nitrofuran Activity

in Multiple Species

Linna Zhou, Hironori Ishizaki, Michaela Spitzer, Kerrie L. Taylor, Nicholas D. Temperley, Stephen L. Johnson, Paul Brear, Philippe Gautier, Zhiqiang Zeng, Amy Mitchell, Vikram Narayan, Ewan M. McNeil, David W. Melton, Terry K. Smith, Mike Tyers, Nicholas J. Westwood, and E. Elizabeth Patton

Inventory of Supplemental Information

1. Supplementary Figures

**Figure S1. Melanocyte sensitivity to NFN1 treatment.
Related to Figure 1.**

**Figure S2. ALDH genes in humans, zebrafish, yeast and trypanosomes.
Related to Figures 2, 3, 5 and 6.**

**Figure S3. Chemical control of the ALDH2-PKC pathway in zebrafish.
Related to Figure 3.**

**Figure S4. 5-Nitrofurans in multiple species.
Related to Figure 4.**

2. Supplementary Tables

**Table S1. Peptide sequences identified by tandem mass spectrometry.
Related to Figure 2.**

3. Supplemental Experimental Procedures and Information for Chemistry

4. Supplementary Movie

**Movie S1. Time-lapse imaging of 5-nitrofuran treatment of live zebrafish.
Related to Figure 1.**

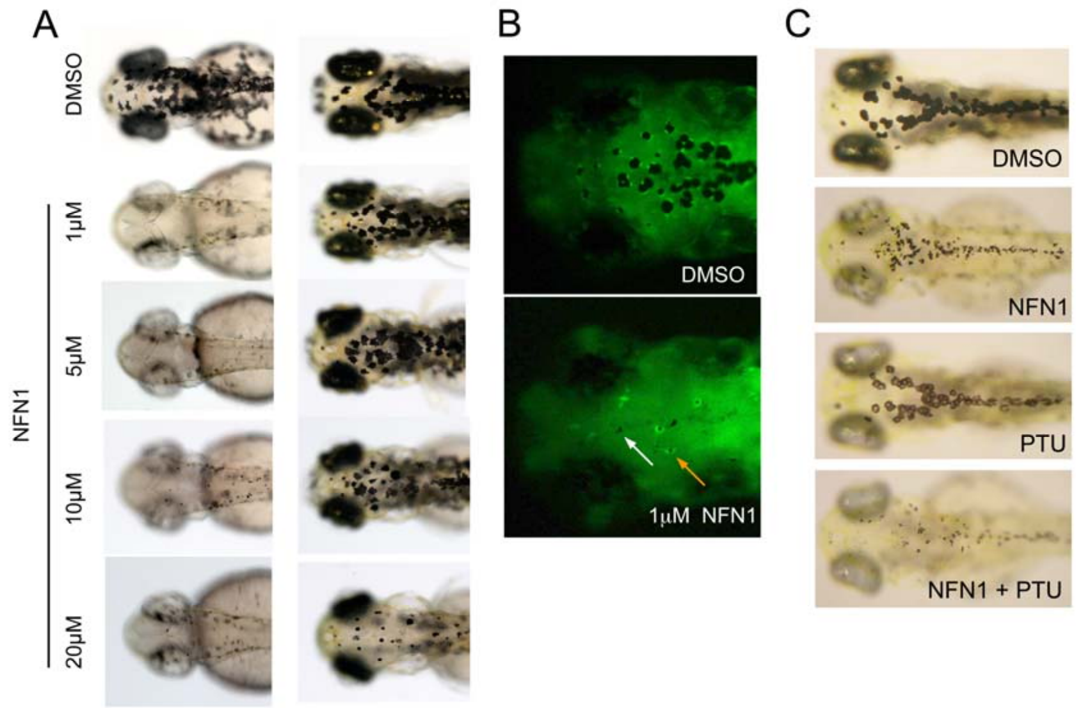

Zhou et al. Fig. S1

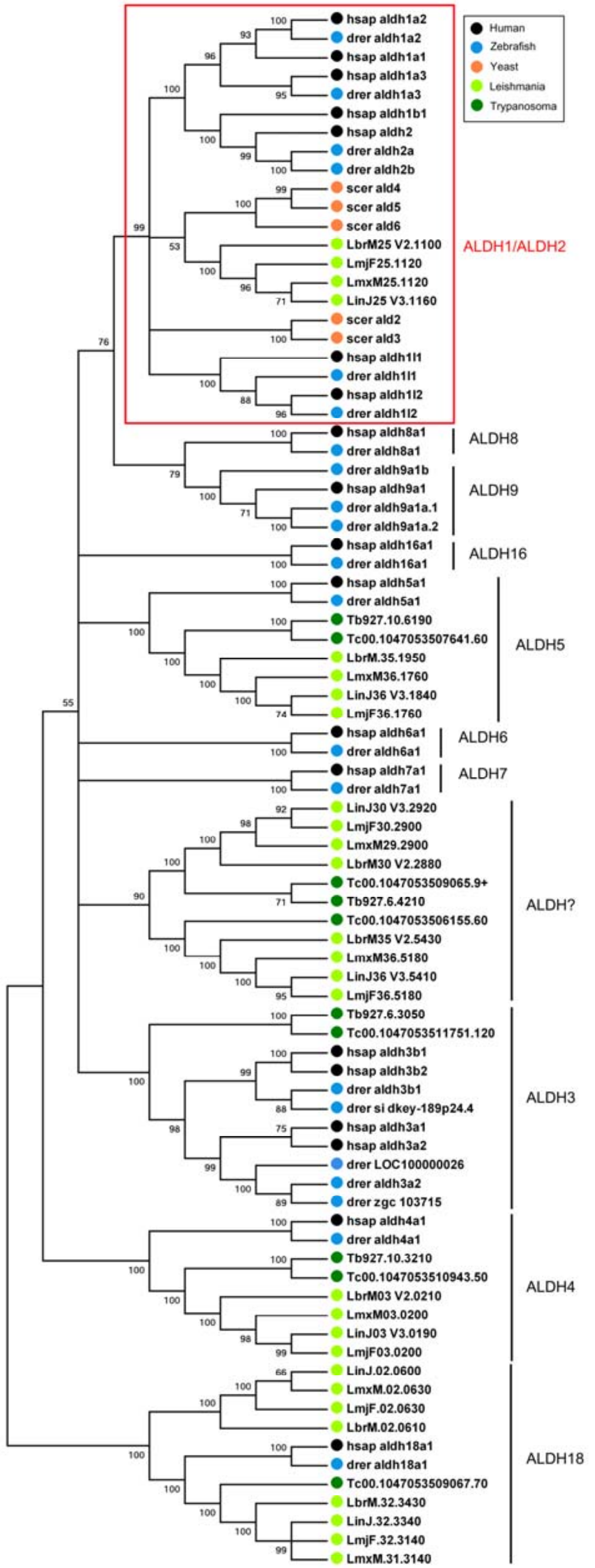

Zhou et al. Fig. S2

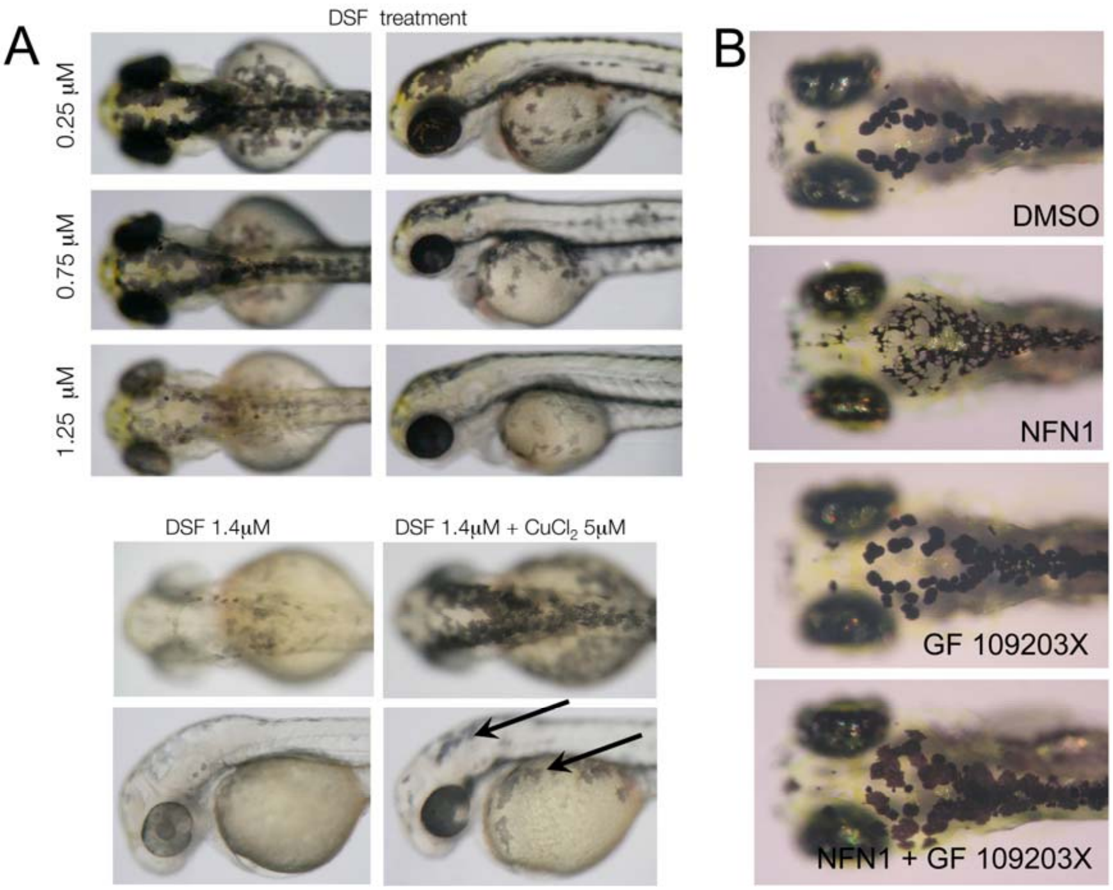

Zhou et al. Fig. S3

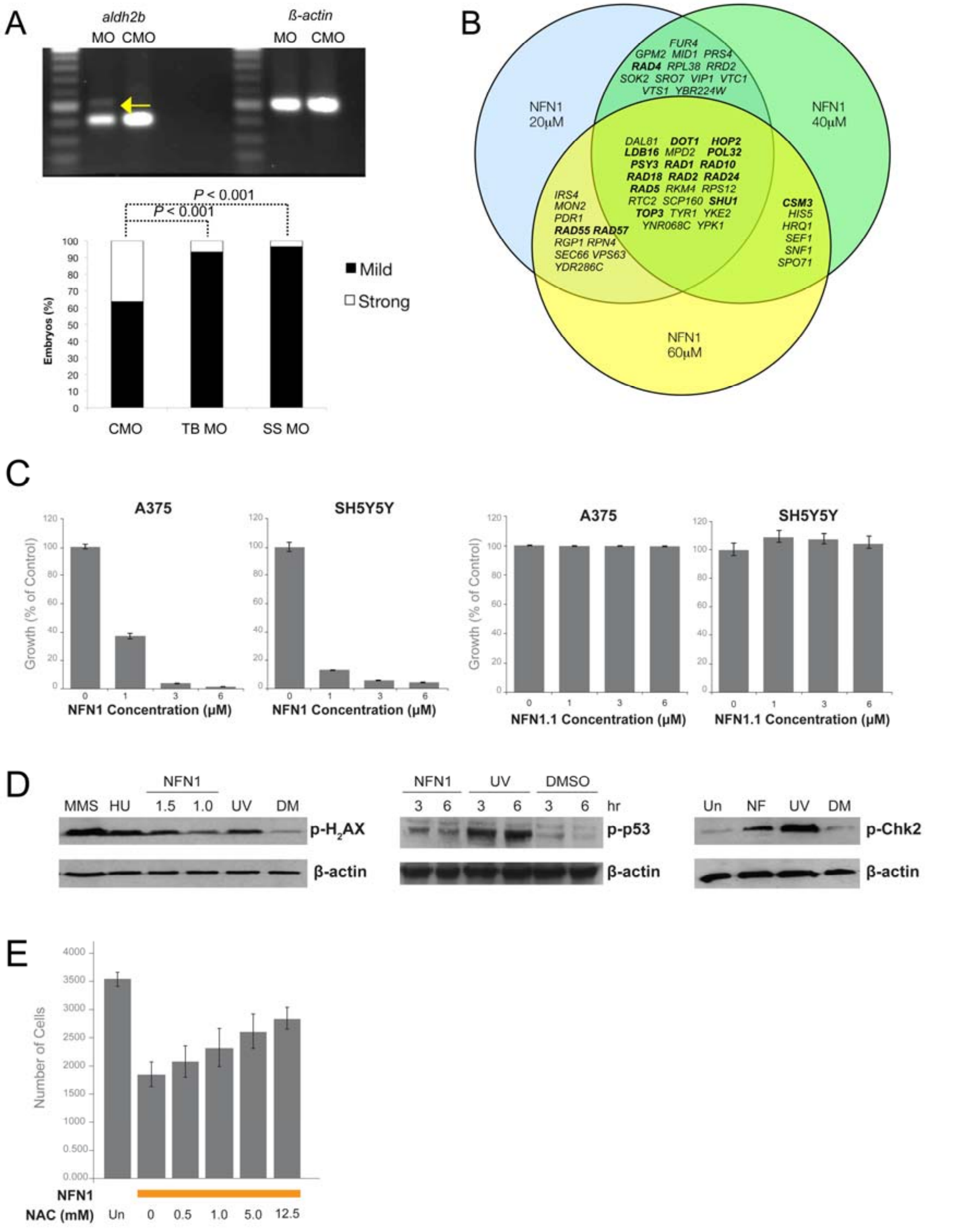

Zhou et al., Fig. S4

Supplementary Figure Legends

Figure S1. **Melanocyte sensitivity to NFN1 treatment. A.** Example images of zebrafish following increasing concentrations of NFN1 (left panel), and transfer to fresh E3 embryo medium (washout) for three days. **B**. Melanocytes are visible in transgenic zebrafish embryos expressing melanocyte specific *tryp1-GFP* both by the black pigment and GFP poistive cytoplasm. In NFN1 treated embryos most melanocytes are no longer visible, and black detritus is not associated with GFP positive cytoplasm (white arrow), although a few melanocytes survive (orange arrow). **C.** PTU does not rescue NFN1 activity in melanocytes. PTU inhibits the activity of tyrosinase, the rate-limiting enzyme required for pigment synthesis. Two dpf zebrafish embryos were pre-treated with 30mg/l PTU for 6 hours, and then treated with 0.5 μM NFN1 or DMSO as a control.

Figure S2. ALDH genes in humans, zebrafish, yeast and trypanosomes. A. A neighbour-joining phylogenetic tree assigns possible ALDH genes in budding yeast and *Trypanosomatidae* to specific ALDH gene family, where possible (hsap: *Homo sapiens*, drer: *Danio rerio*, scer: *Saccharomyces cerevisiae*, Lbr: *Leishmania braziliensis*, Lin: *Leishmania infantum*, Lmj: *Leishmania major*, Lmx *Leishmania mexicana*, Tc: *Trypanosoma cruzi*, Tb: *Trypanosoma brucei*). The tree was rooted using ALDH18 as an outgroup. In Leishmania, four genes are clear orthologs of human ALDH 4, 5, and 18, one belongs to the ALDH1/2 group and two genes form a group together with other Trypanosoma genes which we could not easily assign to any human gene. In Trypanosoma, clear orthologs of human ALDH3, 4, 5 and 18 (*T. cruzi* only) were found while the ALDH1/2 related gene was absent. All yeast genes seem to belong to the ALDH1/2 group.

Figure S3. Chemical control of the ALDH2-PKC pathway in zebrafish . A. Two-day old embryos treated with low doses of DSF maintain pigmentation (top panels). Loss of pigmentation in DSF treated embryos can be restored with copper (lower panels). Pigmented melanocytes are indicated (arrow). **B**. The PKC inhibitor, GF 109203X prevents NFN1-induced phenotypes. Zebrafish embryos grown at 28.5°C for 2 days and treated with NFN1 for 24 hours in the presence of DMSO, NFN1, GF 109203X (Enzo

Life Sciences, UK) or NFN1 and GF 109203X (n=40). Co-treatment with 20μM GF 109203X prevented NFN1 activity in 35/40 treated embryos. Experiments were repeated four times. **C**. Daidzin alters background adaptation in zebrafish embryos. (Left) Images of fixed zebrafish embryos (5 dpf) treated with 0.1% DMSO or 10 μM daidzin, and shifted from a dark environment to a light environment (light), or *vice versa* (dark). The average percentage of melanin coverage (within the area indicated by red dotted outline) for each treatment condition ± standard deviation is indicated. (Right) Box plot of melanin coverage (y-axis) for each embryo in different treatment conditions (x-axis). Individual values taken from one of three experiments are shown as red circles. The box depicts the lower quartile and the upper quartile, with the median depicted by the intersecting line. Whiskers extend between the minimum and maximum of all the data. In DMSO treated embryos, melanocytes are significantly contracted in the light and expanded in the dark (P<0.001, n=20 for each condition); one-way analysis of variance (ANOVA), [mean and 95% confidence interval of differences; 11.081(5.966,16.195)]. Zebrafish treated with daidzin contract their melanin in response to light environment, but do not significantly expand their melanin in response to dark environments [95% CI 0.563(-4.552, 5.677)]. The experiment was repeated three separate times with embryos at 5 dpf (n=5-20 embryos per condition) and once at 4 dpf (n= 10 embryos per condition).

Figure S4. 5-Nitrofurans in multiple species. A. PCR analysis of RNA from 3 dpf embryos injected with a standard control MO (CMO; 8 ng) or an *aldh2b* MO (8 ng) targeting an exon 4 splice-site junction (mis-splicing product with additional intron 4: yellow arrow). (Lower panel) Day 2 morphants were treated with 0.8 μM NFN1 for 8 hours and melanocyte morphology assessed (control (n=55), *aldh2b* translation start-site (TL; n=60) or splice-site (SS; n=57) blocking MOs). Embryos were scored as those that had a strong or mild sensitivity to NFN1 (as described in **Fig. 5**). *aldh2b* TL morphants embryos were significantly less sensitive to NFN1 treatment compared to control morphants [P<0.001; 95% CI (0.155,0.439); Fisher's exact test]. *aldh2b* SS morphants were significantly less sensitive to NFN1 treatment compared to control morphants [P<0.001; 95% CI (0.192,0.464); Fisher's exact test]. To take multiple comparisons into account a p-value of <0.017 was considered to be statistically significant. This

experiment was repeated three times. **B.** Yeast haploid mutants sensitive to 20, 40 and 60 μM NFN1. Deletion strains with a Z-score smaller than -2 are listed. NFN1 sensitive mutants that overlap between screens with different concentrations of NFN1 treatment are grouped. Genes known to function in the DNA damage pathways are in bold. **C**. A375 melanoma and SH5Y5Y neuroblastoma cells were treated with increasing concentrations of NFN1 or NFN1.1 for five days and cell growth measured by sulphorhodamine B (SRB) assay. Mean growth relative to untreated cells (+/- S.E.M.) is shown (n=3). **D**. Western blot analysis of A375 cell lysates for DNA damage response proteins after treatment with NFN1, UV-irradiation, methyl methanesulfonate (MMS), hydroxyurea (HU) or DMSO. Immunoblotting was performed with anti-phospho-H2AX, anti-phospho-p53 (ser15), and anti-phospho-Chk2 (Thr 68) antibodies. **E**. A375 cells were treated with NFN1 (10μM) in the presence of increasing concentrations of the free radical scavenger *N*-acetyl-l-cysteine (NAC) (Calbiochem) for 3 hours, and then analyzed by SRB analysis (standard deviation is indicated; n>3).

Table S1. Peptide sequences identified by tandem mass spectrometry of Aldh 2b [Danio rerio] (NCBI gi|51571951)

**Matched peptides: total coverage 17%** (start – end AA)	**MS/MS Confirmation AA sequence**	**MASCOT score**[1]
195 - 208	LGPALATGNTVVMK (Oxidation M)	56
441 - 454	YGLAGAVFTQNIDK	66
38 - 50	IFINNEWHDAVSK	79
492 - 505	EMGEYGLENYTEVK	71
324 - 337	TFVQESIYDEFVER	71
346 - 367	IVGDPFDLNTEQGPQVNEDQFK	58

[1] Ions score is -10*Log(P), where P is the probability that the observed match is a random event.
Individual ions scores > 54 indicate identity or extensive homology (p<0.05).
Protein scores are derived from ions scores as a non-probabilistic basis for ranking protein hits.

Supplementary Methods and Information for Chemistry

Zebrafish melanin distribution assay. Zebrafish embryos were first kept in the dark for at least five minutes to standardize their environmental light exposure. Forty zebrafish embryos were then transferred to the dark, and forty remained in the light for one hour at room temperature (about 24°C). Half of each cohort was then treated with 10μM daidzin (in 0.1% DMSO) or 0.1% DMSO alone and transferred to the opposite light condition for 30 min. Embryos were then fixed (4% paraformaldehyde) and melanin distribution was quantified in a standardized defined area of the zebrafish head region (S1).

Phylogenetic analysis. Ensembl (release 61, February 2011) and TriTrypDB (version 2.5, November 2010) (S2) databases were used to obtain ALDH sequences. Positions in alignments containing gaps were omitted from subsequent analyses. The phylogenetic tree was constructed by the neighbour-joining method based on the proportion of amino acid sites at which sequences compared were different. The reliability of each interior branch of a given topology was assessed using the bootstrap interior branch test with 1000 bootstrap. Phylogenetic trees were constructed using MEGA (version 4; http://www.megasoftware.net). Percentage identity tables were calculated from the Clustalw alignment, using the Genedoc package with the percentage calculated over the length of the longest sequence.

Supplementary Information for Chemistry

Chemicals and reagents were obtained from either Aldrich or Alfa-Aesar. All reactions involving moisture sensitive reagents were performed in oven dried glassware under a positive pressure of argon. Dichloromethane (DCM) was obtained dry from a solvent purification system (MBraun, SPS-800).

Melting points were recorded in open capillaries using an Electrothermal 9100 melting point apparatus. Infrared spectra were recorded on a Perkin Elmer Spectrum GX FT-IR spectrometer using thin films on KBr discs Absorption maxima are reported as wavenumbers (cm^{-1}) and intensities are quoted as strong (s), medium (m), weak (w) and

broad (br). Low resolution (LR) and high resolution (HR) electrospray mass spectral (ES-MS) analyses were acquired by electrospray ionisation (ESI) within the School of Chemistry, University of St Andrews. Low and high resolution ESI MS were carried out on a Micromass LCT spectrometer or at a high performance orthogonal acceleration reflecting TOF mass spectrometer, coupled to a Waters 2975 HPLC. Nuclear magnetic resonance (NMR) spectra were acquired either on a Bruker Avance 300 (^{1}H, 300.1 MHz; ^{13}C, 75.5 MHz) or on a Bruker Avance 400 (^{1}H, 400 MHz; ^{13}C, 100.1 MHz) spectrometer and in the deuterated solvent stated. ^{13}C NMR spectra were acquired using the PENDANT or DEPTQ pulse sequences. All NMR spectra were acquired using the deuterated solvent as the lock. Coupling constants (*J*) are quoted in Hz and are recorded to the nearest 0.1 Hz. The following abbreviations are used; s, singlet; d, doublet; dd, doublet of doublets; ddd, doublet of doublet of doublets; dt, doublet of triplets; t, triplet; m, multiplet; q, quartet; qt, quintet; br, broad.

1. Synthesis of nitrofuran analogues

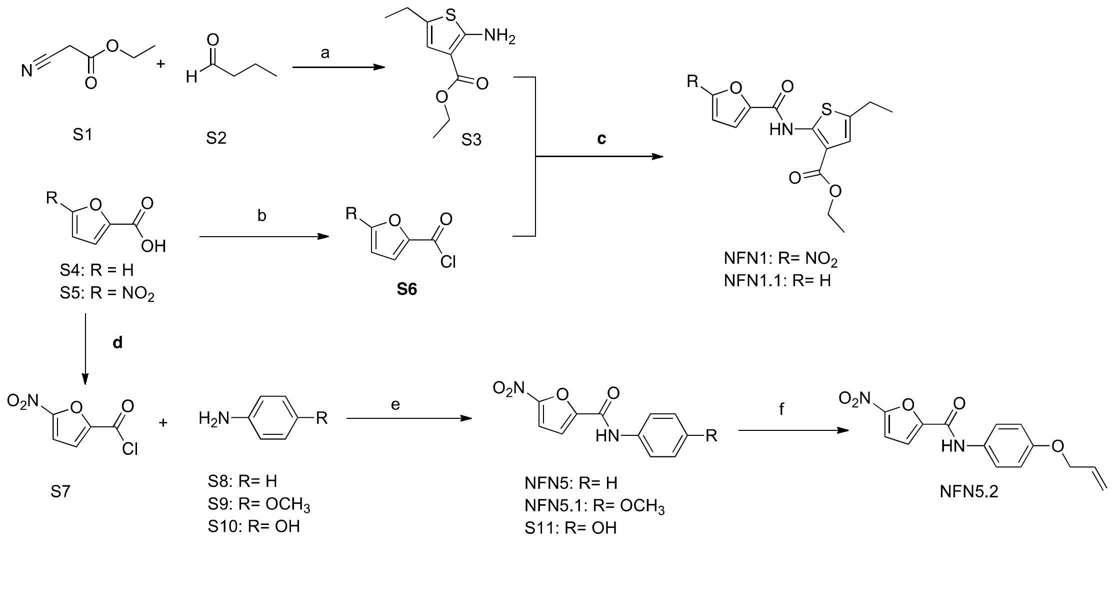

(a) S_8, Et_2NH, DMF, r.t., 4 h, 66%; (b) **S4**, $SOCl_2$, DCM, Et_3N, r.t., 3 h; (c) DCM, Et_3N, r.t., 5 h, 68% (**NFN1**) and 56% (**NFN1.1**) for two steps; (d) **S5**, $SOCl_2$, DCM, Et_3N, r.t., 3 h; (e) aniline **S6**-**8**, DCM, Et_3N, r.t., 5 h, two steps: 61% (**S9**), 51% (**S10**), 69% (**S11**) ; (d) allyl bromide, K_2CO_3, acetone, reflux 2 h, 72%.

Ethyl 2-amino-5-ethylthiophene-3-carboxylate (S3)[S3]

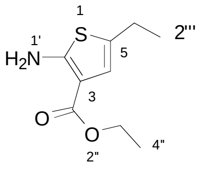

Diethylamine (0.520 mL, 5.00 mmol) was added dropwise to a mixture of α-cyanoester **S1** (commercially available, 0.530 mL, 5.00 mmol), butyraldehyde **S2** (commercially available, 0.450 mL, 5.00 mmol) and sulfur (commercially available, 162 mg, 5.25 mmol) in DMF (5.00 mL). The reaction mixture was stirred at room temperature and monitored by TLC. After complete consumption of the starting material had occurred, the reaction mixture was diluted with ethyl acetate (30.0 mL) and washed with water (3×10.0 mL). The organic layer was dried (Na_2SO_4) and concentrated under reduced pressure. The residue was purified by flash column chromatography (1:15, EtOAc/Hexane) to afford **S3** as a light orange solid (655 mg, 3.20 mmol, 66%). Mp 65-66 ^{o}C (Ref.[S3] 73 ^{o}C); ^{1}H NMR (300 MHz, $CDCl_3$): δ 6.56 (t, ^{4}J= 1.2 Hz, 1H, C4-H), 4.18 (q, ^{3}J= 7.1 Hz, 2H, C3''-H_2), 2.54 (dq, ^{3}J= 7.5 Hz, ^{4}J= 1.2 Hz, 2H, C1'''-H_2), 1.26 (t, ^{3}J= 7.1 Hz, 3H, C4''-H_3), 1.15 (t, ^{3}J= 7.5 Hz, 3H, C2'''-H_3); LRMS (ES^+): m/z (%) 200.31 (100) $[M+H]^+$. The discrepancy between the observed and the literature melting point could not be explained. ^{1}H NMR and low resolution mass spectral analysis were consistent with the desired known product having been prepared.

Ethyl 5-ethyl-2-(furan-2-carboxamido)thiophene-3-carboxylate (NFN1.1)

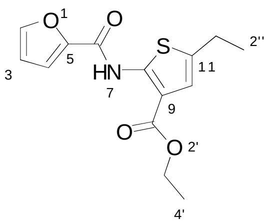

Thionyl chloride (0.140 mL, 1.20 mmol) was added dropwise to a mixture of furan-2-carboxylic acid **S4** (commercially available, 136 mg, 1.20 mmol) and triethylamine (0.210 mL, 1.50 mmol) in anhydrous DCM (10.0 mL) under a N_2 atmosphere. The reaction mixture was stirred at room temperature for 3 hours to give a crude sample of the

chloride **S6,** which was then transferred without further purification to another flask containing ethyl 2-amino-5-ethylthiophene-3-carboxylate **S3** (199 mg, 1.00 mmol) and triethylamine (0.280 mL, 2.00 mmol) in DCM (20.0 mL). The reaction mixture was stirred at room temperature for 5 hours. The solvent was then removed under reduced pressure and the crude product was purified by column chromatography on silica gel (1:7, EtOAc/Hexane) to afford **NFN1.1** as a yellow solid (164 mg, 0.56 mmol, 56%). Mp 100-101 ^{o}C; IR (KBr) ν_{max} = 3411 (m) (NH), 1688 (s) (C=O), 1658 (s) (C=O), 1558 (s), 1275 (s), 1225 (s), 738 (s) cm^{-1}. ^{1}H NMR (400 MHz, d_6-Acetone): δ 11.60 (br. s, 1H, N7-H), 7.76 (dd, ^{3}J= 1.8 Hz, ^{4}J= 0.7 Hz, 1H, C2-H), 7.18 (dd, ^{3}J= 3.6 Hz, ^{4}J= 0.7 Hz, 1H, C4-H), 6.80 (t, ^{4}J= 1.1 Hz, 1H, C10-H), 6.61 (dd, $^{3}J_1$= 3.6 Hz, $^{3}J_2$= 1.8 Hz, 1H, C3-H), 4.24 (q, ^{3}J= 7.1 Hz, 2H, C3'-H$_2$), 2.64 (dq, ^{3}J= 7.5 Hz, ^{4}J= 1.1 Hz, 2H, C1"-H$_2$), 1.25 (t, ^{3}J= 7.1 Hz, 3H, C4'-H$_3$).1.15 (t, ^{3}J= 7.5 Hz, 3H, C2"-H$_3$). ^{13}C NMR (100 MHz, d_6-Acetone): δ 166.0 (C), 154.9 (C), 153.4 (C), 147.5 (C), 147.0 (CH), 146.8 (C), 138.1(C), 120.2 (CH), 117.1 (CH), 113.7 (CH), 61.4 (CH$_2$), 23.2 (CH$_2$), 16.0 (CH$_3$), 14.6 (CH$_3$). LRMS (ES^{+}): m/z (%) 315.90 (100) [M+Na]$^{+}$; HRMS (ES^{+}): m/z calcd for $C_{14}H_{15}NO_4NaS$ [M+Na]$^{+}$: 316.0619; found 316.0613.

General Procedure I: Synthesis of nitrofuran amides

Thionyl chloride (1.20 eq.) was added dropwise to a mixture of 5-nitrofuran-2-carboxylic acid **S5** (commercially available, 1.00 eq.), triethylamine (1.50 eq.) in DCM (0.400 M) under a N_2 atmosphere. The reaction mixture containing crude acid chloride **S7** was stirred at room temperature for 3 hours and then added to another flask containing the corresponding 2-amino-thiophene or aniline (1.00 eq.) and triethylamine (2.00 eq.) in DCM (0.400 M). The reaction mixture was stirred at room temperature for another 5 hours. The solvent was then removed under reduced pressure and the crude product was purified by column chromatography (hexane/ethyl acetate).

Ethyl 5-ethyl-2-(5-nitrofuran-2-carboxamido)thiophene-3-carboxylate (NFN1)

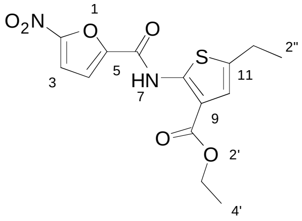

General procedure I was followed using ethyl 2-amino-5-ethylthiophene-3-carboxylate **S3** (3.99 g, 20.0 mmol) to afford **NFN1** as an orange solid (4.60 g, 13.6 mmol, 68%). Mp 127-128 ^{o}C; IR (KBr) ν_{max} = 3120 (s) (NH), 1667 (s) (C=O), 1569 (s) (C=O), 1266 (s), 1256 (s) cm^{-1}. ^{1}H NMR (400 MHz, d_6-Acetone): δ 11.75 (br. s, 1H, NH), 7.58 (d, ^{3}J= 3.9 Hz, 1H, C3-H), 7.42 (d, ^{3}J= 3.9 Hz, 1H, C4-H), 6.85 (t, ^{4}J= 1.1 Hz, 1H, C10-H), 4.28 (q, ^{3}J= 7.1 Hz, 2H, C3'-H$_2$), 2.67 (dq, ^{3}J= 7.5 Hz, ^{4}J= 1.1 Hz, 2H, C1"-H$_2$), 1.27 (t, ^{3}J= 7.1 Hz, 3H, C4'-CH$_3$), 1.17 (t, ^{3}J= 7.5 Hz, 3H, C2"-H$_3$). ^{13}C NMR (100 MHz, d_6-Acetone): δ 165.8 (C), 153.6 (C), 153.1 (C), 147.4 (C), 145.6 (C), 139.2 (C), 120.6 (CH), 118.6 (CH), 115.0 (C), 113.8 (CH), 61.7 (CH$_2$), 23.2 (CH$_2$), 15.9 (CH$_3$), 14.6 (CH$_3$). LRMS (ES^{+}): m/z (%) 360.87 (100) [M+Na]$^{+}$; HRMS (ES^{+}): m/z calcd for $C_{14}H_{14}N_2O_6NaS$ [M+Na]$^{+}$: 361.0470; found 361.0469.

5-nitro-*N*-phenylfuran-2-carboxamide (NFN5)[S4, S3]

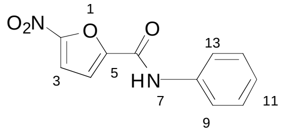

General procedure I was followed using aniline **S8** (186 mg, 2.00 mmol) to afford **NFN5** as a yellow solid (283 mg, 1.22 mmol, 61%). Mp 174-175 ^{o}C (Ref [S3] 174-177 ^{o}C); ^{1}H NMR (300 MHz, d_6-Acetone): δ 9.94 (br. s, 1H, NH), 7.70 (d, ^{3}J= 8.2 Hz, 2H, C9-H, C13-H), 7.50 (d, ^{3}J= 3.9 Hz, 1H, C3-H), 7.33 (d, ^{3}J= 3.9 Hz, 1H, C4-H), 7.24 (dd, $^{3}J_1$= 8.2 Hz, $^{3}J_2$= 7.4 Hz, 2H, C10-H, C12-H), 7.03 (t, ^{3}J= 7.4 Hz, 1H, C11-H); LRMS (ES^{-}): m/z (%) 231.06 (100) [M-H]$^{-}$. Three pieces of analytical data have been provided to support the synthesis of the known compound **NFN5**.

***N*-(4-methoxyphenyl)-5-nitrofuran-2-carboxamide (NFN5.1)**[S6, S7]

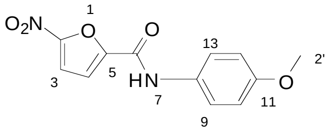

General procedure I was followed using 4-methoxyaniline **S9** (615 mg, 5.00 mmol) to afford **NFN5.1** as a yellow solid (670 mg, 2.56 mmol, 51%). Mp: 184-185 °C (Ref [S7] 183 °C); IR (KBr) ν_{max} = 3372 (s) (NH), 1678 (s) (C=O), 1515 (s), 1259 (s). ^{1}H NMR (400 MHz, d_6-Acetone): δ 9.86 (br s, 1H, N7-H), 7.61 (d, ^3J= 7.9 Hz, 2H, C9-H, C13-H), 7.51 (d, ^3J= 3.9 Hz, 1H, C3-H), 7.31 (d, ^3J= 3.9 Hz, 1H, C4-H), 6.81 (d, ^3J= 7.9 Hz, 2H, C10, C12-H), 3.67 (s, 3H, C2'-H_3); LRMS (ES$^-$): m/z (%) 261.01 (100) [M-H]$^-$. Four pieces of analytical data have been provided to support the synthesis of the known compound **NFN5.1**.

***N*-(4-hydroxyphenyl)-5-nitrofuran-2-carboxamide (S11)** [S6, S7]

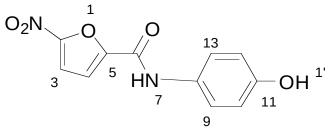

General procedure I was followed using 4-amino-phenol **S10** (959 mg, 8.00 mmol) to afford **S11** as an orange solid (1.37g, 5.52 mmol, 69%). Mp: Dec.> 262 °C (Ref [S6] 267 °C); ^{1}H NMR (400 MHz, d_6-Acetone): δ 9.89 (br. s, 1H, NH), 8.33 (s, 1H, OH), 7.59 (d, ^3J= 9.0 Hz, 2H, C9-H, C13-H), 7.58 (d, ^3J= 3.9 Hz, 1H, C3-H), 7.38 (d, ^3J= 3.9 Hz, 1H, C4-H), 6.79 (d, ^3J= 9.0 Hz, 2H, C10-H, C12-H); LRMS (ES$^-$): m/z (%) 247.05 (100) [M-H]$^-$. Three pieces of analytical data have been provided to support the synthesis of the known compound **S11**.

***N*-(4-(allyloxy)phenyl)-5-nitrofuran-2-carboxamide (NFN5.2)**

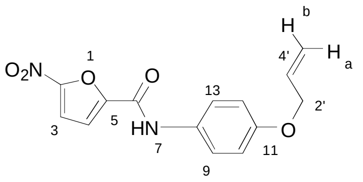

A mixture of *N*-(4-hydroxyphenyl)-5-nitrofuran-2-carboxamide **S11** (80 mg, 0.32 mmol, 1.00 eq.), K_2CO_3 (88 mg, 0.64 mmol, 2.00 eq.) and allyl bromide (0.060 mL, 0.64 mmol, 2.00 eq.) in acetone (5.0 mL) was refluxed for 2 hours. After cooling, the reaction mixture was diluted in ethyl acetate (20.0 mL), washed with water (10.0 mL), dried (Na_2SO_4), and concentrated under reduced pressure. The crude product was purified by flash column chromatography over silica gel (1:4, EtOAc/hexane) to afford **NFN5.2** as an orange solid (66 mg, 0.23 mmol, 72%). Mp: 152-153 ^{o}C; IR (KBr) ν_{max} = 3316 (m) (NH), 1664 (s) (C=O), 1541 (N=O), 1513 (N=O), 1349 (s), 811 (s) cm^{-1}. ^{1}H NMR (400 MHz, d_6-Acetone): δ 9.88 (br s, 1H, N7-H), 7.61 (dd, ^{3}J= 6.5 Hz, ^{4}J= 2.2 Hz, 2H, C13-H, C9-H), 7.51 (d, ^{3}J= 3.8 Hz, 1H, C3-H), 7.31 (d, ^{3}J= 3.8 Hz, 1H, C4-H), 6.83 (d, ^{3}J= 6.5 Hz, 2H, C10-H, 12-H), 5.93 (m, 1H, C3'-H), 5.29 (d, ^{3}J= 18.0 Hz, 1H, C4'-Ha), 5.11 (d, ^{3}J= 10.5 Hz, 1H, C4'-Hb), 4.67 (m, 2H, C2'-H_2). ^{13}C NMR (100 MHz, d_6-Acetone): δ 156.0 (C), 155.5 (C), 152.1 (C), 148.9 (C), 135.1 (CH), 132.5 (C), 123.3 (2CH), 117.8 (CH_2), 117.4 (CH), 116.0 (2CH), 114.1 (CH), 69.9 (CH_2). LRMS (ES$^-$): m/z (%) 287.04 (100) [M-H]$^-$; HRMS (ES$^-$): m/z calcd for $C_{14}H_{11}N_2O_5$ [M-H]$^-$: 287.0668; found 287.0662.

2. Chemical probes used in affinity matrix

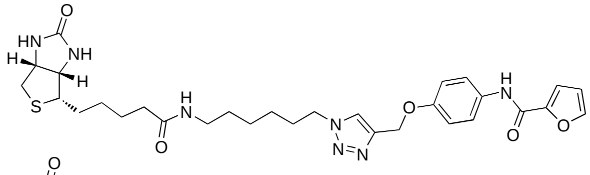

Control probe (Pr-FN)

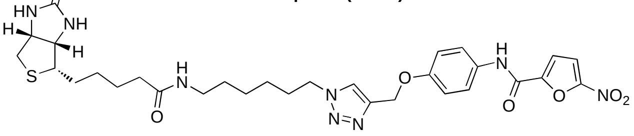

Active probe (Pr-NFN)

3. Synthesis of the chemical probes

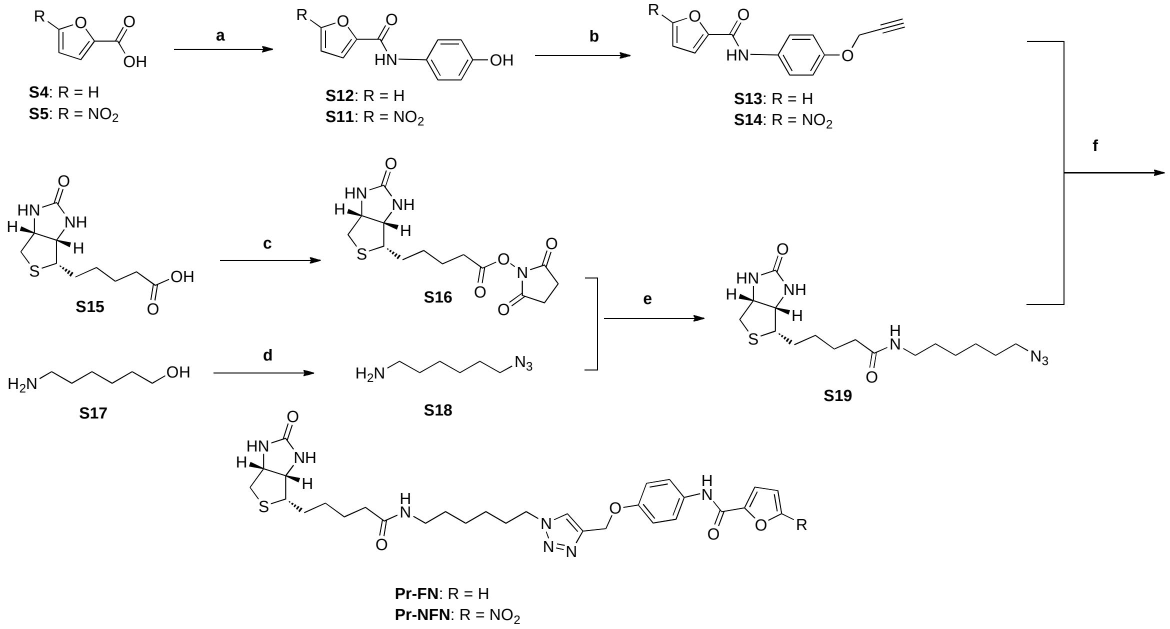

(a) i) $SOCl_2$, DCM, Et_3N, r.t., 3 h; ii) DCM, Et_3N, r.t., 5 h, **S12** (53%), **S11** (69%); (b) K_2CO_3, acetone, **S13**: propargyl bromide (2 eq.), rt, 24 hrs, 86%; **S14**: propargyl bromide (1 eq.), reflux, 3 hrs, 46%; (c) DCC, DMF, rt, o/n, 74%; (d) i): $SOCl_2$, toluene, reflux, 1h; ii): NaN_3, water, 90 °C, 2 hrs, 71%; (e) NEt_3, MeOH, rt, o/n, 85%; (f) NaASc (2.8 eq.), $CuSO_4$ $5H_2O$ (1.4 eq.), ^{t}BuOH/H_2O (1:2), 48 hrs, **Pr-FN** (61%), **Pr-NFN** (59%).

N-(4-hydroxyphenyl)furan-2-carboxamide (S12) [S8]

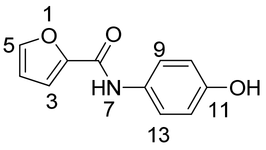

General procedure I was followed using furan-2-carboxylic acid **S4** (3.36 g, 30.0 mmol) and 4-amino-phenol **S10** (2.19 mL, 30.0 mmol) to afford **S12** as a white solid (3.21 mg, 15.8 mmol, 53%). Mp: Dec>223 °C (Ref [S8] 218-219 °C); ^{1}H NMR (400 MHz, d_6-DMSO): δ 9.95 (br. s, 1H, N7-H), 9.29 (br. s, 1H, O-H), 7.88 (d, ^3J= 1.8 Hz, 1H, C5-H), 7.47 (d, ^3J= 8.8 Hz, 2H, C9-H, C13-H), 7.24 (d, ^3J= 3.5 Hz, 1H, C3-H), 6.71 (d, ^3J= 8.8 Hz, 2H, C10-H, C12-H), 6.66 (dd, $^3J_1$= 3.5 Hz, $^3J_2$= 1.8 Hz, 1H, C4-H); LRMS (ES$^+$): m/z (%) 204.32 (100) [M+H]$^+$. Three pieces of analytical data have been provided to support the synthesis of the known compound **S12**.

N-(4-(prop-2-yn-1-yloxy)phenyl)furan-2-carboxamide (S13)

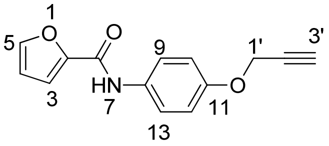

A mixture of **S12** (250 mg, 1.23 mmol), K_2CO_3 (339 mg, 2.46 mmol) and propargyl bromide (0.192 mL, 2.46 mmol) in acetone (5 mL) was stirred at room temperature for 24 hours. The reaction mixture was then diluted with ethyl acetate (20.0 mL), washed with water (10.0 mL), dried (Na_2SO_4) and concentrated under *vacuo*. The crude was purified by column chromatography over silica gel (3:1, hexane/ethyl acetate) to afford **S13** as a light brown solid (254 mg, 1.05 mmol, 86%). Mp 84-85 °C; IR ν_{max} = 3282 (m) (NH), 3108 (w), 2120 (w) (alkynyl), 1666 (m) (C=O), 1510 (s), 1208 (m), 1012 (m), 854 (m) cm^{-1}. ^1H NMR (400 MHz, d_6-Acetone): δ 9.21 (br. s, 1H, N7-H), 7.64 (dt, ^3J= 9.0 Hz, ^4J= 2.0 Hz, 2H, C9-H, C13-H), 7.60 (d, ^3J= 1.8 Hz, 1H, C5-H), 7.05 (d, ^3J= 3.6 Hz, 1H, C3-H), 6.88 (dt, ^3J= 9.0 Hz, ^4J= 2.0 Hz, 2H, C10-H, C12-H), 6.50 (dd, ^3J_a= 3.6 Hz, ^3J_b= 1.8 Hz, 1H, C4-H), 4.65 (d, ^4J= 2.4 Hz, 2H, C1'-H_2), 3.03 (d, ^4J= 2.4 Hz, 1H, C3'-H). ^{13}C NMR (100 MHz, d_6-Acetone): δ 156.9 (C), 155.1 (C), 149.3 (C), 145.5 (CH), 133.3 (C), 122.5 (2CH), 115.9 (2CH), 115.1 (CH), 112.9 (CH), 79.0 (CH), 76.9 (C), 56.6 (CH_2). LRMS (ES^+): m/z (%) 263.82 (100) $[M+Na]^+$; HRMS (ES^+): m/z calcd for $C_{14}H_{11}NO_3Na$ $[M+Na]^+$: 264.0637; found 264.0642.

5-nitro-N-(4-(prop-2-yn-1-yloxy)phenyl)furan-2-carboxamide (S14)

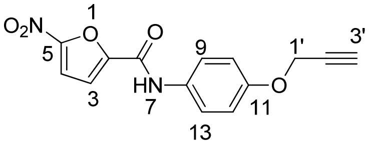

The phenol compound **S11** (496 mg, 2.00 mmol), propargyl alcohol (176 μL, 3.00 mmol), PPh_3 (787 mg, 3 mmol) were dissolved in dry THF (15.0 mL). DAED (476 μL, 3.00 mmol) was added slowly to the reaction mixture, and then stirred at room temperature overnight. The solvent was then removed and the residue was participated

between EtOAc and water. The organic layer was dried, concentrated in vacuo and purified with column chromatography (1:4, EtOAc/Hexane) to afford **S14** as an orange solid (265 mg, 0.926 mmol, 46%). Mp: 127-128 °C; IR ν_{max} = 3365 (m), 3273 (m) (NH), 3183 (w), 2120 (w) (alkynyl), 1685 (m) (C=O), 1510 (s) (N=O), 1340 (m) (N=O), 1230 (s), 821 (s) cm^{-1}. ^{1}H NMR (400 MHz, d_6-Acetone): δ 9.89 (br s, 1H, N7-H), 7.64 (dt, ^3J= 9.0 Hz, ^4J= 2.0 Hz, 2H, C9-H, C13-H), 7.56 (d, ^3J= 3.8 Hz, 1H, C4-H), 7.31 (d, ^3J= 3.8 Hz, 1H, C3-H), 6.90 (dt, ^3J= 9.0 Hz, ^4J= 2.0 Hz, 2H, C10-H, C12-H), 4.67 (d, ^4J= 2.4 Hz, 2H, C1'-H_2), 3.03 (d, ^4J= 2.4 Hz, 1H, C3'-H). ^{13}C NMR (100 MHz, d_6-Acetone): δ 155.6 (C), 155.2 (C), 152.8 (C), 149.6 (C), 132.7 (C), 122.9 (2CH), 117.0 (CH), 115.9 (2CH), 113.7 (CH), 79.8 (C), 77.0 (CH), 56.5 (CH_2). LRMS (ES^+): m/z (%) 308.95 (100) $[M+Na]^+$; HRMS (ES^+): m/z calcd for $C_{14}H_{10}N_2O_5Na$ $[M+Na]^+$: 309.0486; found 309.0487.

2,5-dioxopyrrolidin-1-yl 5-((3a*S*,4*S*,6a*R*)-2-oxohexahydro-1H-thieno[3,4-d]imidazol-4-yl) pentanoate (S16) [S9, S10]

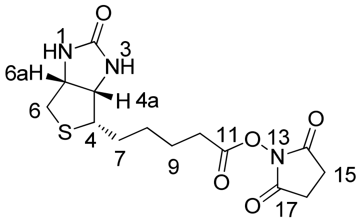

Biotin **S15** (1g, 4.09 mmol, 1.00 eq.) was dissolved in dry DMF (20 mL) at 80 °C. After cooling down, hydroxyl succinimide (612 mg, 5.32 mmol, 1.3 eq.) and DCC (888 mg, 4.09 mmol, 1.00 eq.) were added to the reaction mixture, which was stirred at room temperature overnight. After completion of the reaction, DCU was filtrated and the filtrate was concentrated *in vacuo*. The residue was then washed with boiled 2-propanol (10 mL), and dried to provide **S16** as a white solid (1.03 g, 3.03 mmol, 74%). Mp 196-197 °C (Ref [S10] 201-202 °C); ^{1}H NMR (300 MHz, d_6-DMSO): δ 6.41 (br. s, 1H, N1-H), 6.35 (br. s, 1H, N3-H), 4.31-4.28 (m, 1H, C6a-H), 4.15-4.12 (m, 1H, C4a-H), 3.10-3.08 (m, 1H, C4-H), 2.88-2.81 (m, 5H, C15-H_2, C16-H_2, C6-Ha), 2.68 (t, ^3J= 14.8 Hz, 2H, C10-H_2), 2.56 (d, ^2J= 12.4 Hz, 1H, C6-Hb), 1.65-1.40 (m, 6H, C7-H_2, C8-H_2, C9-H_2); LRMS (ES^+): m/z (%) 342.44 (100) $[M+H]^+$. Three pieces of analytical data have been provided to support the synthesis of the known compound **S16**.

6-azidohexan-1-amine (S18) [S9]

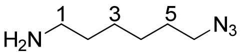

A mixture of 6-amino-hexan-1-ol **S17** (1.17 g, 10.0 mmol, 1.00 eq.) and $SOCl_2$ (3.28 mL, 45.0 mmol, 4.50 eq.) in toluene (10.0 mL) was refluxed for 1 hour. After cooling down, the solvent was removed *in vacuo*. The 6-chloro-1-hexylamine intermediate was used directly in next step without purification. Sodium azide (1.95 g, 30.0 mmol, 3.00 eq.) and water (10.0 mL) was added. The reaction mixture was then stirred at 90 ^{o}C for 2 hours, before being basified with KOH to pH (12-14) and then extracted with DCM, dried ($NaSO_4$) and concentrated *in vacuo*. The product **S18** was obtained as a off white liquid (1.01 g, 7.10 mmol, 71%). ^1H NMR (300 MHz, $CDCl_3$): δ 3.20 (t, ^3J= 6.9 Hz, 2H, C6-H_2), 2.63 (t, ^3J= 6.9 Hz, 2H, C1-H_2), 1.57-1.28 (m, 8H, 4CH_2); LRMS (ES^+): m/z (%) 143.25 (100) $[M+H]^+$. Two pieces of analytical data have been provided to support the synthesis of the known compound **S18**.

***N*-(6-azidohexyl)-5-((3a*S*,4*S*,6a*R*)-2-oxohexahydro-1H-thieno[3,4-d]imidazol-4-yl)pentanamide (S19)** [S9]

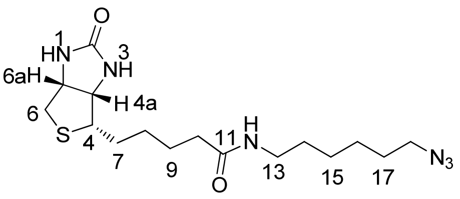

Biotin succinimidyl ester **S16** (500 mg, 1.46 mmol) was added slowly to a mixture of 6-azido-1-hexylamine **S18** (312 mg, 2.19 mmol) and NEt_3 (0.31 mL, 2.19 mmol) in methanol (30.0 mL). The reaction mixture was stirred at room temperature for overnight. The solvent was then removed *in vacuo* and the residue was purified by column chromatography (10% methanol/DCM). The product **S19** was obtained as a pale yellow solid (457 mg, 1.24 mmol, 85%). Mp: 151-152 ^{o}C; ^1H NMR (300 MHz, d_6-DMSO): δ 7.73 (t, ^3J= 5.4 Hz, 1H, N12-H), 6.42 (br. s, 1H, N1-H), 6.35 (br. s, 1H, N3-H), 4.33-4.28 (m, 1H, C6a-H), 4.12-4.10 (m, 1H, C3a-H), 3.31 (t, ^3J= 6.9 Hz, 2H, C18-H_2), 3.10-3.06 (m, 1H, C4-H), 3.04-2.98 (m, 2H, C13-H_2), 2.82 (dd, ^2J= 12.6 Hz, ^3J= 5.1 Hz, 1H, C6-Ha), 2.56 (d, ^2J= 12.6 Hz, 1H, C6-Hb), 2.04 (t, ^3J= 7.3 Hz, 2H, C10-H_2), 1.81-1.23 (m,

14H, $7CH_2$); LRMS (ES^+): m/z (%) 369.54 (100) $[M+H]^+$. No literature melting point is available for **S19**. Two pieces of analytical data have been provided to support the synthesis of the known compound **S19**.

General procedure II: Click chemistry

Biotin azide **S19** (1.00 mmol, 1.00 eq.), the corresponding alkynyl compound (1.10 mmol, 1.10 eq.), sodium ascorbate (2.80 mmol, 2.80 eq.) and $CuSO_4.5H_2O$ (1.40 mmol, 1.40 eq.) were added to a solution of ^{t}BuOH/H_2O (10 mL/20 mL) and the reaction mixture was stirred at room temperature for 48 hours. More than one equivalent of copper salt was needed. The solvent was then removed *in vacuo* and the residue was purified by column chromatography (1:9, methanol/DCM) to afford the required product.

***N*-(4-((1-(6-(5-((3a*S*,4*S*,6a*R*)-2-oxohexahydro-1*H*-thieno[3,4-d]imidazol-4-yl)pentanamido)hexyl)-1*H*-1,2,3-triazol-4-yl)methoxy)phenyl)furan-2-carboxamide (Pr-FN)**

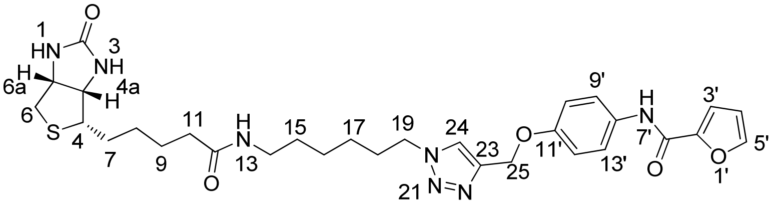

General procedure II was followed using biotin azide **S19** (353 mg, 0.96 mmol), compound **S13** (254 mg, 1.05 mmol). The product **Pr-FN** was obtained as a white solid (356 mg, 0.58 mmol, 61%). Mp 178-179 °C; IR ν_{max} = 3466 (w), 3320 (br) (NH), 2972 (m), 2879 (w), 1687 (s) (C=O), 1543 (s) (C=O), 1530 (s), 1251 (s) (C-O), 1023 (w), 835 (m), 754 (m) cm^{-1}. ^{1}H NMR (400 MHz, d_6-DMSO): δ 10.10 (br. s, 1H, N7'-H), 8.23 (s, 1H, C24-H), 7.91 (dd, ^3J= 1.8 Hz, ^4J= 0.7 Hz, 1H, C5'-H), 7.74 (t, ^3J= 5.5 Hz, 1H, N13-H), 7.65 (d, ^3J= 9.1 Hz, 2H, C9'-H, C13'-H), 7.29 (dd, ^3J= 3.5 Hz, ^4J= 0.7 Hz, 1H, C3'-H), 7.01 (d, ^3J= 9.1 Hz, 2H, C10'-H, C12'-H), 6.89 (dd, ^3J_a= 3.5 Hz, ^3J_b= 1.8 Hz, 1H, C4'-H), 6.44 (br, 1H, N1-H), 6.37 (br, 1H, N3-H), 5.11 (s, 2H, C25-H_2), 4.38-4.28 (m, 3H, C19-H_2, C6a-H), 4.12-4.09 (m, 1H, C4a-H), 3.11-3.05 (m, 1H, C4-H), 3.03-2.97 (m, 2H, C14-H_2), 2.81 (dd, ^2J= 12.6 Hz, ^3J= 5.1 Hz, 1H, C6-Ha), 2.57 (d, ^2J= 12.6 Hz, 1H,

C6-Hb), 2.04 (t, ^{3}J= 7.3 Hz, 2H, C11-H$_2$), 1.81-1.23 (m, 14H, 7CH$_2$). ^{13}C NMR (100 MHz, d_6-DMSO): δ 171.7 (C), 162.6 (C), 155.9 (C), 154.3 (C), 147.6 (C), 145.4 (CH), 142.6 (C), 131.8 (C), 124.3 (CH), 121.9 (2CH), 114.6 (2CH), 114.3 (CH), 112.0 (CH), 61.2 (CH), 61.0 (CH$_2$), 59.1 (CH), 55.4 (CH), 49.3 (CH$_2$), 39.4 (CH$_2$), 38.2 (CH$_2$), 35.2 (CH$_2$), 29.61 (CH$_2$), 28.9 (CH$_2$), 28.2 (CH$_2$), 28.0 (CH$_2$), 25.7 (CH$_2$), 25.5 (CH$_2$), 25.3 (CH$_2$). LRMS (ES$^+$): m/z (%) 631.78 (100) [M+Na]$^+$; HRMS (ES$^+$): m/z calcd for $C_{30}H_{39}N_7O_5NaS$ [M+Na]$^+$: 632.2631; found 632.2631.

5-nitro-*N*-(4-((1-(6-(5-((3a*S*,4*S*,6a*R*)-2-oxohexahydro-1*H*-thieno[3,4-d]imidazol-4-yl)pentanamido)hexyl)-1*H*-1,2,3-triazol-4-yl)methoxy)phenyl)furan-2-carboxamide (Pr-NFN)

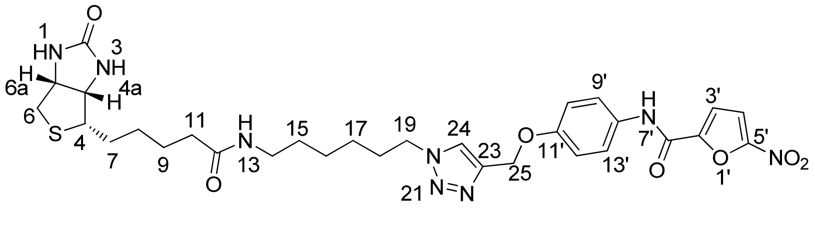

General procedure II was followed using biotin azide **S19** (585 mg, 1.56 mmol) and compound **S14** (500 mg, 1.75 mmol). The product **Pr-NFN** was obtained as an orange solid (609 mg, 0.93 mmol, 59%). Mp 201-202 ^{o}C; IR ν_{max} =3273 (br) (NH), 2922 (m), 2864 (w), 1697 (s) (C=O), 1520 (s) (N=O), 1363 (m) (N=O), 1260 (s) (C-O), 1055 (w), 825 (m) cm^{-1}. ^{1}H NMR (400 MHz, d_6-DMSO): δ 10.55 (br. s, 1H, N7'-H), 8.23 (s, 1H, C24-H), 7.81 (d, ^{3}J= 3.9 Hz, 1H, C3'-H), 7.73 (t, ^{3}J= 5.4 Hz, 1H, N13-H), 7.65 (d, ^{3}J= 9.0 Hz, 2H, C9'-H, C13'-H), 7.60 (d, ^{3}J= 3.9 Hz, 1H, C4'-H), 7.06 (d, ^{3}J= 9.0 Hz, 2H, C10'-H, C12'-H), 6.42 (br, 1H, N1-H), 6.36 (br, 1H, N3-H), 5.13 (s, 2H, C25-H$_2$), 4.35 (t, ^{3}J= 7.1 Hz, 2H, C19-H$_2$), 4.30-4.28 (m, 1H, C6a-H), 4.14-4.10 (m, 1H, C4a-H), 3.12-3.07 (m, 1H, C4-H), 3.03-2.97 (m, 2H, C14-H$_2$), 2.81 (dd, ^{2}J= 12.5 Hz, ^{3}J= 5.0 Hz, 1H, C6-Ha), 2.57 (d, ^{2}J= 12.5 Hz, 1H, C6-Hb), 2.04 (t, ^{3}J= 7.3 Hz, 2H, C11-H$_2$), 1.83-1.23 (m, 14H, 7CH$_2$). ^{13}C NMR (100 MHz, d_6-DMSO): δ 171.8 (C), 162.7 (C), 154.9 (C), 154.2 (C), 151.6 (C), 148.1 (C), 142.5 (C), 131.0 (C), 124.3 (CH), 122.2 (2CH), 116.2 (CH), 114.8 (2CH), 113.5 (CH), 61.2 (CH), 61.0 (CH$_2$), 59.1 (CH), 55.4 (CH), 49.3 (CH$_2$), 39.4 (CH$_2$), 38.2 (CH$_2$), 35.2 (CH$_2$), 29.61 (CH$_2$), 28.9 (CH$_2$), 28.2 (CH$_2$), 28.0

(CH_2), 25.7 (CH_2), 25.5 (CH_2), 25.3 (CH_2). LRMS (ES^+): m/z (%) 676.87 (100) $[M+Na]^+$; HRMS (ES^+): m/z calcd for $C_{30}H_{38}N_8O_7NaS$ $[M+Na]^+$: 677.2482; found 677.2486.

HPLC analysis of purity

HPLC analysis was performed using a Gilson UV-VIS 155 HPLC system under the gradient conditions shown in the analysis method below (**Table 3**) (RP, reverse phase), XTerra RP 18 5 µm column (3.0 x 50 mm, Waters). The concentration of the compounds were *ca.* 4 mM, injection volumes were 20 µL, flow rate was 1 mL/min and detection was acquired using UV spectroscopy (254 nm)

HPLC analysis method

Time (min)	%H_2O^a	% CH_3CN
0	80	20
10	20	80
15	20	80
17	80	20
20	80	20

[a]With 0.1% TFA

Retention times and purities of new compounds

Compound	t_r (min)	Purity
**NFN1**	7.71	>98%
**NFN1.1**	7.55	>98%
**NF5**	3.68	>98%
**NFN5.1**	3.61	>98%
**NFN5.2**	5.22	>98%
**S13**	3.53	>98%
**S14**	4.73	>98%
**Pr-FN**	4.49	>97%
**Pr-NFN**	3.67	>98%

HPLC and MS analysis of nifurtimox

Analytical RP-HPLC was performed on an analytical Agilent HP1100 system with ELSD detector equipped with a Phenomenex Prodigy C18 reverse phase column (100 x 4.6 mm i.d.), eluting with a gradient of water/formic acid (0.1%) to MeOH/formic acid (0.1%) in 10 minutes. Analysis was carried out with the 30mM solution of Nifurtimox (source: Dr. G. Sholler, Van Andel Institute, USA) in DMSO used in the experiments. HPLC analysis showed an early peak (before minute 1, consistent with DMSO) and a main peak at 5.030 min, consistent with Nifurtimox chemical polarity[S11] and purity of >95%. HPLC/ELSD: 5.030 min.

Low-resolution mass spectra were recorded on a VG Platform Quadrupole Electrospray Ionisation mass spectrometer. Nifurtimox m/z signal was observed (310 = Nifurtimox + Na+; also observed in Prestwick sample). Due to the excess of DMSO, the main peak observed in the MS was m/z 101 (corresponding to the MW of DMSO plus Na+). MS (ES+): m/z (%): 101.0 (100) [DMSO + Na]+ , 310.0 (9) [Nifurtimox + Na]+.

Purchased Compound Suppliers and Purity

Compound	Supplier	Purity
BTB05727 (NFN1)	Maybridge / in house source	>90%
SEW00138 (NFN2)	Maybridge	>90%
BTB13657 (NFN3)	Maybridge	>90%
BR00087 (NFN4)	Maybridge	>90%
Daidzin	Sigma-Aldrich	>95%
PKC-412	Enzo Lifesciences	>98%
Ro31 8220	Enzo Lifesciences	>98%
GF109203X	Enzo Lifesciences	>98%
PTU	Sigma-Aldrich	>98%
Disulfiram	Sigma-Aldrich	>97%
Nifurtimox	Bayer Argentina	>95%

Supplementary References

S1. Richardson J*, et al.* (2008) mc1r Pathway regulation of zebrafish melanosome dispersion. *Zebrafish* 5(4):289-295.

S2. Aslett M*, et al.* (2010) TriTrypDB: a functional genomic resource for the Trypanosomatidae. *Nucleic Acids Res* 38(Database issue):D457-462 .

S3: Kitagawa, T., Kuroda, H., Sasaki, H., Kawasaki, K. Facile conversions of carboxylic acids into amides, esters, and thio esters using 1,1'-oxalyldiimidazole and 1,1'-oxalyldi-1,2,4-triazole. *Chem. Pharm. Bull.* **35**, 4294-4301 (1987).

S4: Tangallapally, R.P., Yendapally, R., Lee, R.E., Hevener, K., Jones, V.C., Lenaerts, A.J.M., McNeil, M.R.; Wang, Y.; Franzblau, S.; Lee, R.E. Synthesis and Evaluation of Nitrofuranylamides as Novel Antituberculosis Agents. *J. Med. Chem.* **47**, 5276-5283 (2004).

S5: Takahashi, T., Saikachi, H., Sasaki, T., Suzuki, K., Moritani, E. Syntheses of furan derivatives. IV. *Yakugaku Zasshi* **69**, 286-289 (1949).

S6: Farcasan, V., Makkay, C. Furan derivatives. I. p-Substituted anilides of 5-nitro-2-furoic acid. *Acad. rep. populare Romine*, *Filiala Cluj, Studii cercetri chim.* **8**, 151-158 (1949).

S7: WSS: Spectral data were obtained from Wiley Subscription Services, Inc. (US).

S8: Jancevska, M.; Jakopcic, K.; Hahn, V. Hydroxythiomides. I. A simple preparation of aromatic and heteroaromatic thiocarbanilides with a free hydroxyl group. *Croatica Chemica Acta* **37**, 67-72 (1965).

S9: Inverarity, I. A.; Viguier, R. F. H.; Cohen, Philip.; Hulme, A. N. Biotinylated Anisomycin: A comparison of classical and "click" chemistry approaches. *Bioconjugate Chem.* **18**, 1593-1603 (2007).

S10: Piggott, A.M., Karuso, P. Rapid identification of a protein binding partner for the marine natural product kahalalide F by using reverse chemical proteomics. *ChemBioChem* **9**, 524-530 (2008).

S11: de la Concepción Foces-Foces, M., Cano, F. H., Claramunt, R. M., Fruchier, A. and Elguero, J. (1988), Molecular structure in the solid state (X-Ray diffraction) and in solution (^{1}H and ^{13}C NMR) of nifurtimox and two pyrazol-1-Yl analogs. Bulletin des Sociétés Chimiques Belges, 97: 1055–1066.

^{1}H NMR of **Pr-FN**

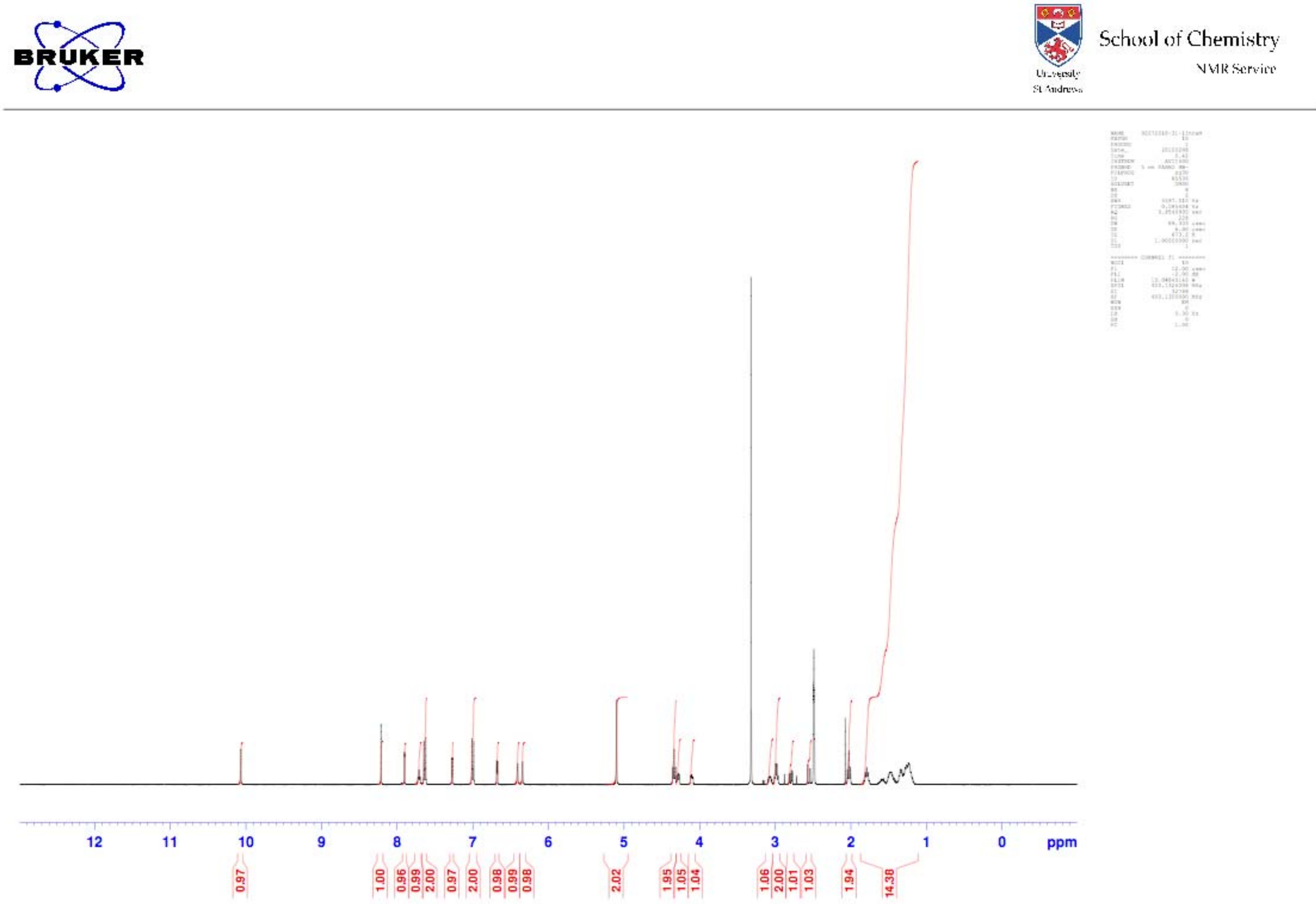

^{13}C NMR of **Pr-FN**

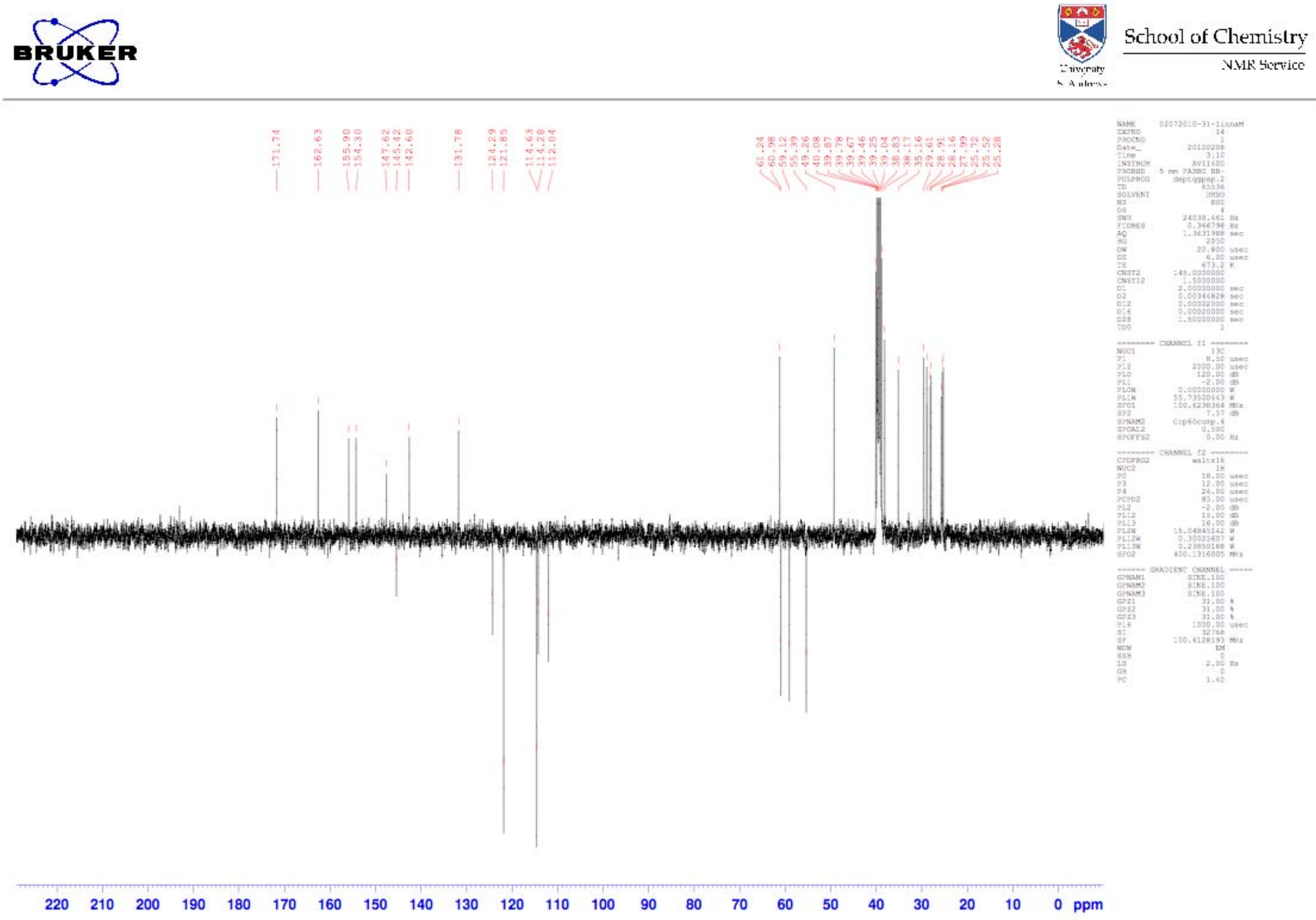

^{1}H NMR of **Pr-NFN**

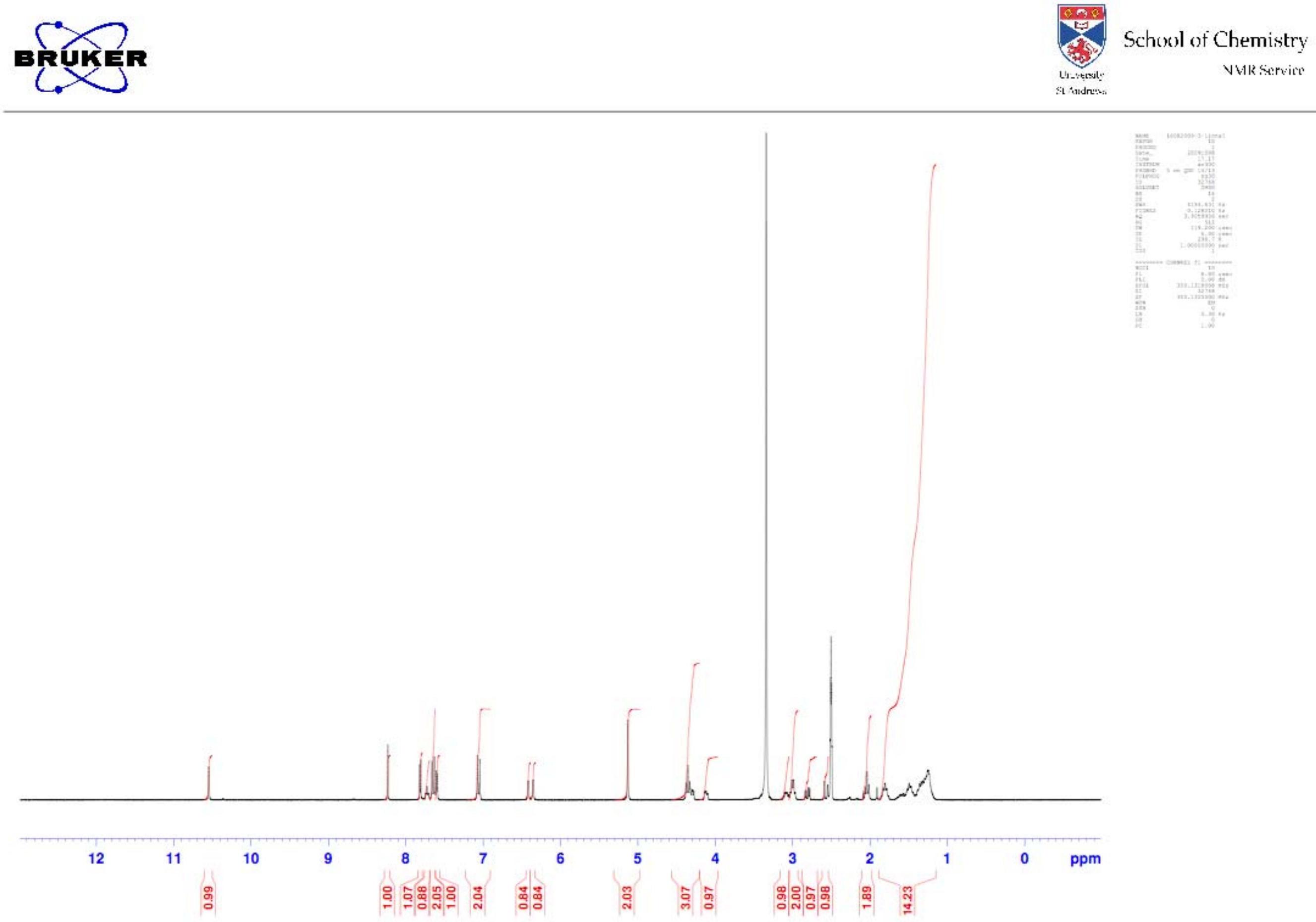

^{13}C NMR of **Pr-NFN**

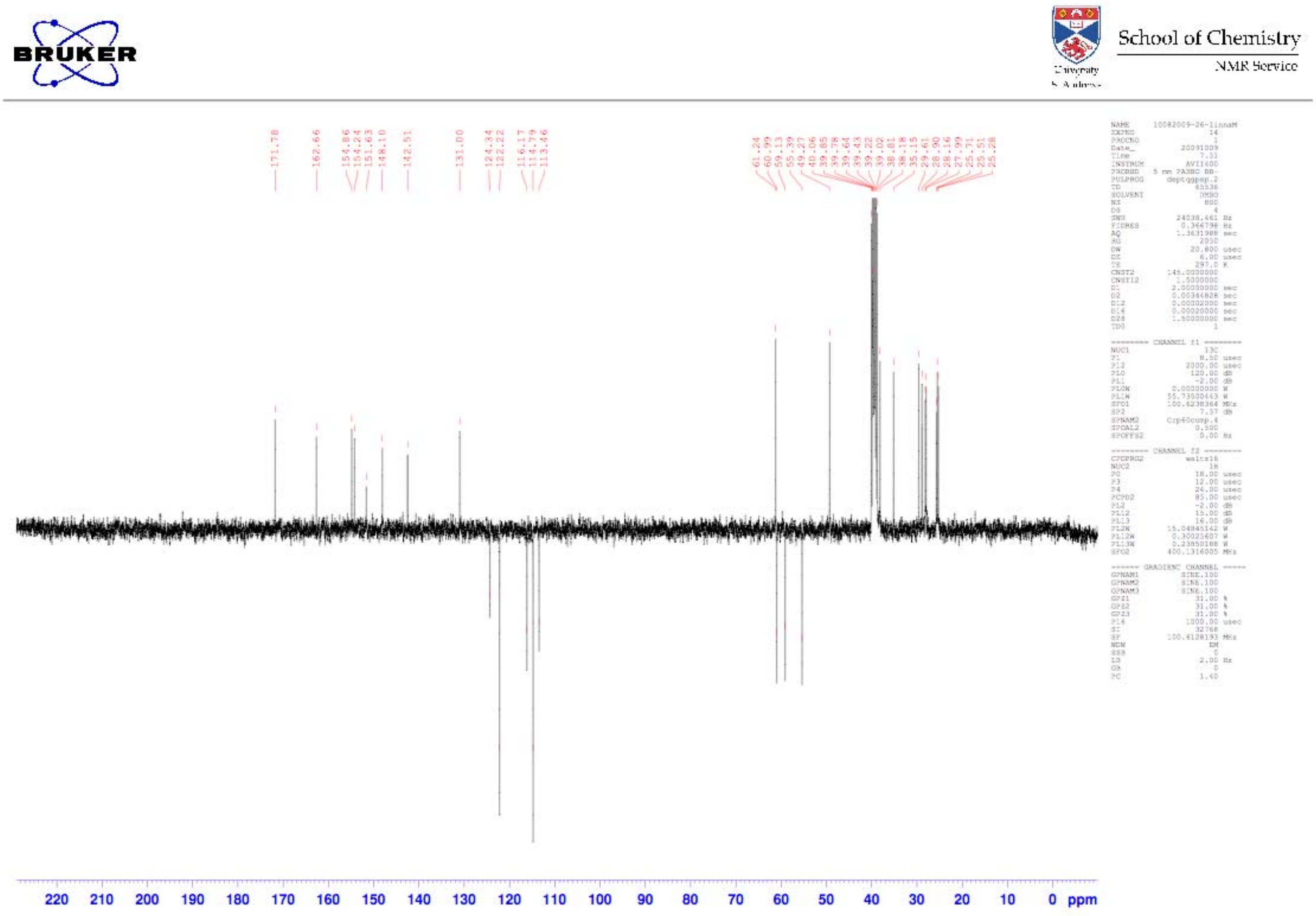

Supplement: Document 1. Figures S1–S4, Table S1, and Supplemental Experimental Procedures [file mmc1.pdf]
