# Supplementary figures and images for: ALDH2 Mediates 5-Nitrofuran Activity in Multiple Species
Source: Chem Biol. 2012 Jul 27;19(7):883–92. doi: 10.1016/j.chembiol.2012.05.017 (PMC3684953; doi:10.1016/j.chembiol.2012.05.017)

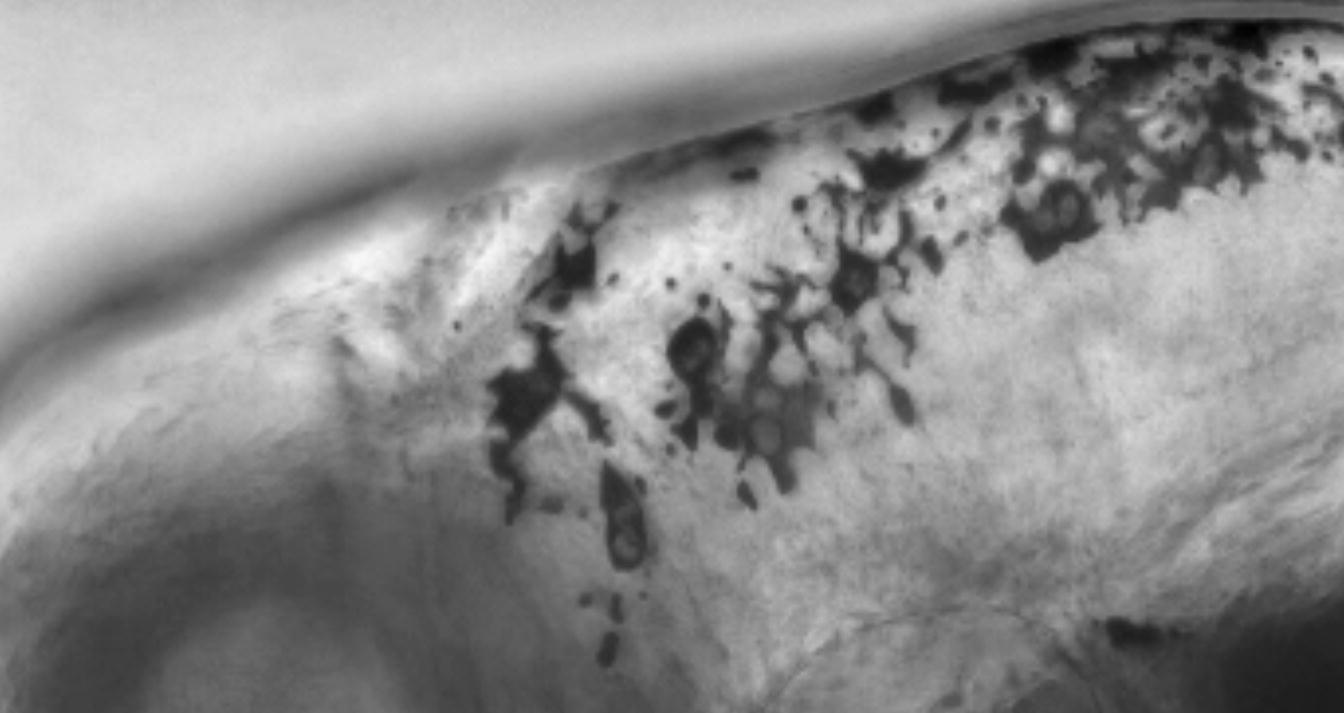

Supplement: Movie S1. Time-Lapse Imaging of 5-Nitrofuran Treatment of Live Zebrafish, Related to Figure 1 [file mmc2.jpg]
